# Elucidating Non-aqueous Solvent Stability and Associated Decomposition Mechanisms for Mg Energy Storage Applications From First-Principles

**DOI:** 10.3389/fchem.2019.00175

**Published:** 2019-04-09

**Authors:** Trevor J. Seguin, Nathan T. Hahn, Kevin R. Zavadil, Kristin A. Persson

**Affiliations:** ^1^Joint Center for Energy Storage Research, Argonne, IL, United States; ^2^Energy Technologies Division, Lawrence Berkeley National Laboratory, Berkeley, CA, United States; ^3^Material, Physical and Chemical Sciences Center, Sandia National Laboratories, Albuquerque, NM, United States; ^4^Department of Materials Science, University of California, Berkeley, Berkeley, CA, United States

**Keywords:** multivalent batteries, electrolytes, density functional theory, decomposition mechanism, bifurcation

## Abstract

Rational design of novel electrolytes with enhanced functionality requires fundamental molecular-level understanding of structure-property relationships. Here we examine the suitability of a range of organic solvents for non-aqueous electrolytes in secondary magnesium batteries using density functional theory (DFT) calculations as well as experimental probes such as cyclic voltammetry and Raman spectroscopy. The solvents considered include ethereal solvents (e.g., glymes) sulfones (e.g., tetramethylene sulfone), and acetonitrile. Computed reduction potentials show that all solvents considered are stable against reduction by Mg metal. Additional computations were carried out to assess the stability of solvents in contact with partially reduced Mg cations (Mg^2+^ → Mg^+^) formed during cycling (e.g., deposition) by identifying reaction profiles of decomposition pathways. Most solvents, including some proposed for secondary Mg energy storage applications, exhibit decomposition pathways that are surprisingly exergonic. Interestingly, the stability of these solvents is largely dictated by magnitude of the kinetic barrier to decomposition. This insight should be valuable toward rational design of improved Mg electrolytes.

## Introduction

**Figure 1:**
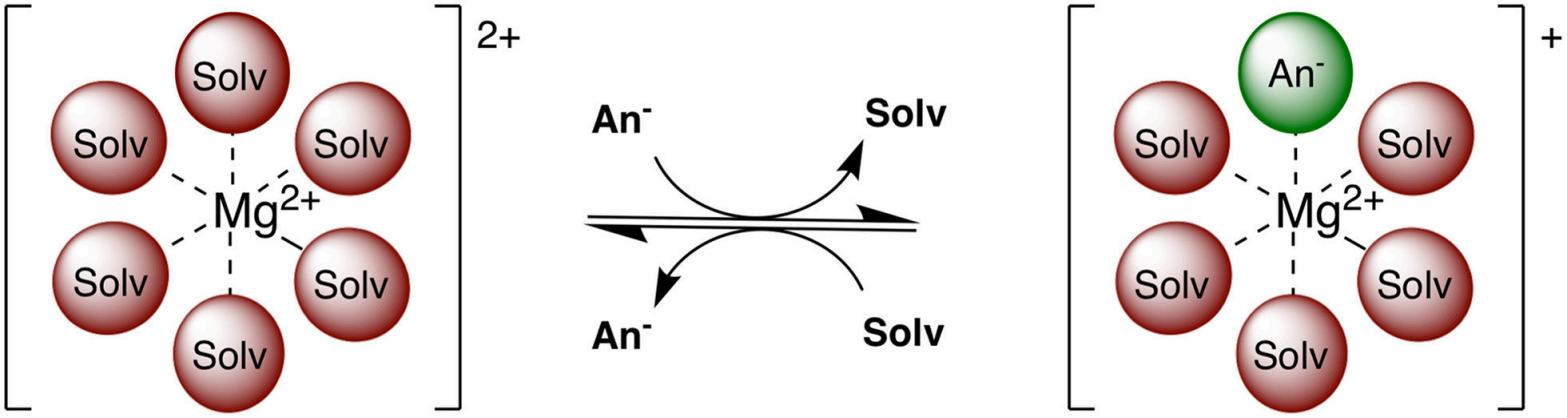
Two examples of possible electroactive species in Mg electrolytes. Among these, the Mg^2+^ complex can adopt an anion in the coordination sphere and retain a positive charge.

Multivalent batteries containing e.g., Mg, Zn, or Ca anodes pose significant improvements to secondary energy storage over the widely implemented Li technology (Mohtadi and Mizuno, 2014; Muldoon et al., 2014). However, the search for suitable electrolytes, exhibiting a wider electrochemical window combined with suitable conductivity, is still ongoing (Muldoon et al., 2012; Song et al., 2016). Electrolyte properties such as conductivity, viscosity, solvation structure, transport, and chemical stability are highly dependent on the exact electrolyte formulation and a complex interplay of interactions between electrolyte species (Rajput et al., 2018). For example, the coordination environment of Mg^2+^ ions consists of solvent and/or anion(s) depending on competing ion-ion and ion-solvent interactions (see Figure 1). Electroactive species containing a divalent cation frequently contain anions in the first solvation shell at moderate concentrations (Rajput et al., 2015), unlike the monovalent cations (e.g., Li^+^), with potential ramifications on electrolyte performance such as conductivity and reversible plating and stripping. Additionally, in non-aqueous electrolytes, only a limited number of solvents are currently practical in terms of their ability to dissociate Mg salts and withstand the plating and stripping process.

Theoretical methods such as Molecular Dynamics (MD) simulations and quantum chemistry calculations have granted invaluable insight to the molecular level effects governing electrolyte behavior (Rajput et al., 2018). For example, it was suggested that the incompatibility of some electrolyte species with Mg metal may be due to reactivity with a transient, partially reduced radical Mg monocation (Mg^2+^ → Mg^+^) formed during the plating process (Rajput et al., 2015). This was first shown for the bis(trifluoromethane)sulfonimide (TFSI) anion which, while having desirable properties such as a relatively high anodic limit, has been associated with poor performance in some electrolyte formulations (particularly at >0.5 M salt concentrations). Theoretical calculations have shown higher ion-association tendency at higher concentrations and an exothermic bond dissociation reaction in the TFSI^−^/Mg^+^ ion-pair, suggesting chemical instability in this situation as an origin of the poor metal plating/deposition reversibility (Rajput et al., 2015).

In addition to compatibility with Mg metal, electrolyte properties such as weak ion-association and high anodic thresholds are desirable. While considerable progress has been made in these areas, it has mostly been derived from a focus on the anion species, and development in regards to the solvent has been less addressed. Some of the most promising recent electrolytes have anodic thresholds that are dictated by the solvent (Tutusaus et al., 2015; Zhao-Karger et al., 2017). Ethereal solvents such as the glymes are perhaps most commonly employed for Mg electrolytes, but their anodic limit is relatively low. In anticipation of electrolyte salts with ever-increasing dissociativity and anodic stability, the burden of chemical and electrochemical stability will increasingly fall on the solvent. Some solvents have highly desirable properties but their practical application in Mg electrolytes is a subject of ongoing investigation. For example, some polar solvents such as sulfones have a relatively high anodic threshold and boiling point, and recent reports have implemented them in various electrolyte formulations with varying degrees of success (Senoh et al., 2014; Kang et al., 2017; Merrill and Schaefer, 2018). One report showed reversibility of an electrolyte formulation of MgCl_2_ in a tetrahydrofuran(THF)/dipropyl sulfone solvent mixture that exhibited reasonable cycling performance after initial conditioning cycles, which required very large over potentials (−1 V vs. Mg) to obtain Mg deposition (Kang et al., 2017). Another report utilizing Mg(HMDS)_2_ and MgCl_2_ dissolved in THF/sulfone mixtures showed conditioning-free cycling and an extent of electrolyte decomposition that was dependent on the sulfone used (Merrill and Schaefer, 2018). Interestingly, using ESI-MS, mixtures of ${\text{Mg}}_{\text{2}}{\text{Cl}}_{3}^{+}$, and MgCl^+^ species were identified and better performance was found in electrolytes with higher ${\text{Mg}}_{\text{2}}{\text{Cl}}_{3}^{+}$ content (Merrill and Schaefer, 2018). Acetonitrile (ACN) is another polar solvent with a relatively high anodic potential limit. However, practical implementation of ACN is hindered, principally, by a reported tendency toward reduction at Mg/Mg^2+^ potentials (Lu et al., 1999; Tran et al., 2012; Shterenberg et al., 2017). Theoretical predictions from MD simulations show, in general, a greater tendency of ion cluster formation in ACN-based electrolytes compared to those of other solvents, with possible implications on ACN's relative ability to dissociate salts (Rajput et al., 2015). However, we note an experimental report that shows good solubility of the MgTFSI_2_ salt in particular with ACN (Veryasov et al., 2016). To our knowledge, the only ACN-based electrolyte documented to show any degree of reversible Mg deposition is a Mg(PF_6_)_2_ salt dissolved in a mixed THF:ACN solvent system (Keyzer et al., 2016). However, the apparent Coulombic efficiency based on the reported voltammetry is very low, indicating poor stability of the electrolyte constituents. These mixed results point to greater reductive susceptibility in the case of ACN than either sulfones or glymes.

Issues related to Mg deposition suggests unsuitable chemical and/or electrochemical stability of the electrolyte. Systematic identification of the source of instability in these systems could lead to insight toward rational design of improved electrolytes. In this report, we examine, using density functional theory (DFT) calculations as well as experimental probes such as cyclic voltammetry and Raman spectroscopy, origins of performance for several organic solvents commonly considered for Mg electrolytes in the case of low ion-pairing, i.e., when the vast majority of Mg cations are only coordinated to solvent. We accomplish this by evaluating stability against reduction by Mg metal for these solvents as well as chemical stability while coordinated to the transient Mg^+^ state likely encountered during initial reduction of the solvated Mg^2+^ cation.

## Experimental Methods

MgTFSI_2_ (99.5%) was purchased from Solvionic and dried under vacuum at 160°C before use. Tetramethylene sulfone (TMS, 99%) was purchased from Sigma-Aldrich and dried by storage over 3A molecular sieves at slightly above the melting point of TMS (~30°C). MgTFSI_2_/TMS solutions were prepared at elevated temperature, after which they were handled at room temperature due to their depressed freezing point. Tetraglyme (G4, 99%) was purchased from Sigma-Aldrich in anhydrous form, distilled, and stored over 3A molecular sieves. Raman spectroscopy was performed using a WITec Confocal Raman Microscope with a 532 nm excitation laser. Raman bands in the TFSI breathing mode region (720–770 cm^−1^) corresponding to free and coordinated TFSI anions were fitted for quantification using a Gaussian/Lorentzian peak fitting method similar to that reported by others (Giffin et al., 2014; Watkins and Buttry, 2015). Two additional vibrational modes corresponding to TMS were also identified in this region from the spectrum of neat TMS, and these were included in addition to the three primary TFSI bands. Electrochemical testing was performed using a beaker cell consisting of Pt working electrode, Mg counter electrode, and Ag/AgBF_4_/3-methylsulfolane reference electrode, calibrated to Mg/Mg^2+^ using a reversible Mg plating solution as demonstrated previously (Hahn et al., 2018). A trace amount of Bu_2_Mg solution was added to the cell to minimize the influence of oxidizing impurities on the Mg plating response in the case of TMS whereas electrochemical pre-conditioning was sufficient in the case of G4 (Shterenberg et al., 2015).

## Theoretical Methods

All calculations were carried out using Gaussian 16 (Frisch et al., 2016). Geometries and vibrational frequencies for free energies involved in Mg^+^-mediated decomposition were computed at the PCM/wB97X-D/6-31G(d) level of theory (Miertuš et al., 1981; Tomasi et al., 2005; Chai and Head-Gordon, 2008). Geometries were characterized as either a potential energy surface minimum (no imaginary frequencies) or a transition state (one imaginary frequency). Final relative free energies are given at the PCM/wB97X-D/6-311+G(d,p)//PCM/wB97X-D/6-31G(d) level of theory—that is, the PCM/wB97X-D method was used with the 6-31G(d) basis set to obtain optimized geometries and free energy thermal corrections, and the thermal corrections were added to single point energies obtained with a larger triple ζ basis set containing a diffuse function and an extra polarization function to obtain final free energies. All free energies are at room temperature (298.15 K). Conversions of free energies to those of different concentrations were made using

$$\text{G}=\text{G}\prime \text{+ RTln}\frac{\text{C}}{\text{C}\prime },$$

where G**′** is the free energy at concentration C**′** (1/24.5 M, equivalent to 1 atm used by Gaussian 16 by the ideal gas law) and G is the free energy at concentration C. For C, 1 M was used for free energies of Mg^+^ species. For free energies of solvent molecules not coordinated to Mg, C was based on the solvent liquid density. Electron affinities were computed under the adiabatic approximation (Ong and Ceder, 2010; Qu et al., 2015) at the PCM/wB97X-D/6-311+G(d,p) level of theory to model the reduction potential of individual and Mg^2+^-coordinated solvent molecules. Difficulties were noted converging the geometry optimization for some transition state structures, requiring a raising of the scaling factor for the van der Waals radii used for construction of the solute cavity from the default (1.1 in Gaussian 16) to a minimum of 1.5. A discussion on the effect of this parameter on the location of the transition state structures is provided in the Supplementary Material. Within the PCM method, a scaling factor of 1.5 was specified for the UFF van der Waals radii used in construction of the solute cavity for the calculations involving Mg^+^-mediated decomposition. For the computed reduction potentials, the default scaling factor was used (1.1). Atomic charges were computed by a natural population analysis (Reed et al., 1985). Molecular graphics were generated using UCSF Chimera (Pettersen et al., 2004). A 3-dimensional contour plot was generated using Paraview (Ayachit, 2015).

## Results and Discussion

The solvents investigated in this study for their role in the Mg electrolyte performance are monoglyme (G1), diglyme (G2), triglyme (G3), dimethyl sulfone (DMS), tetramethylene sulfone (TMS), and ACN. Possible origins of performance issues due to these solvents may arise from poor intrinsic stability against reduction by Mg metal; or in the case of solvents coordinated to Mg^2+^ cations from the bulk electrolyte, poor stability against reduction by coordinated transient Mg^+^ cations, and/or Mg^+^-mediated decomposition. Each of these cases are explored here.

### Reduction Potentials

**Figure 2:**
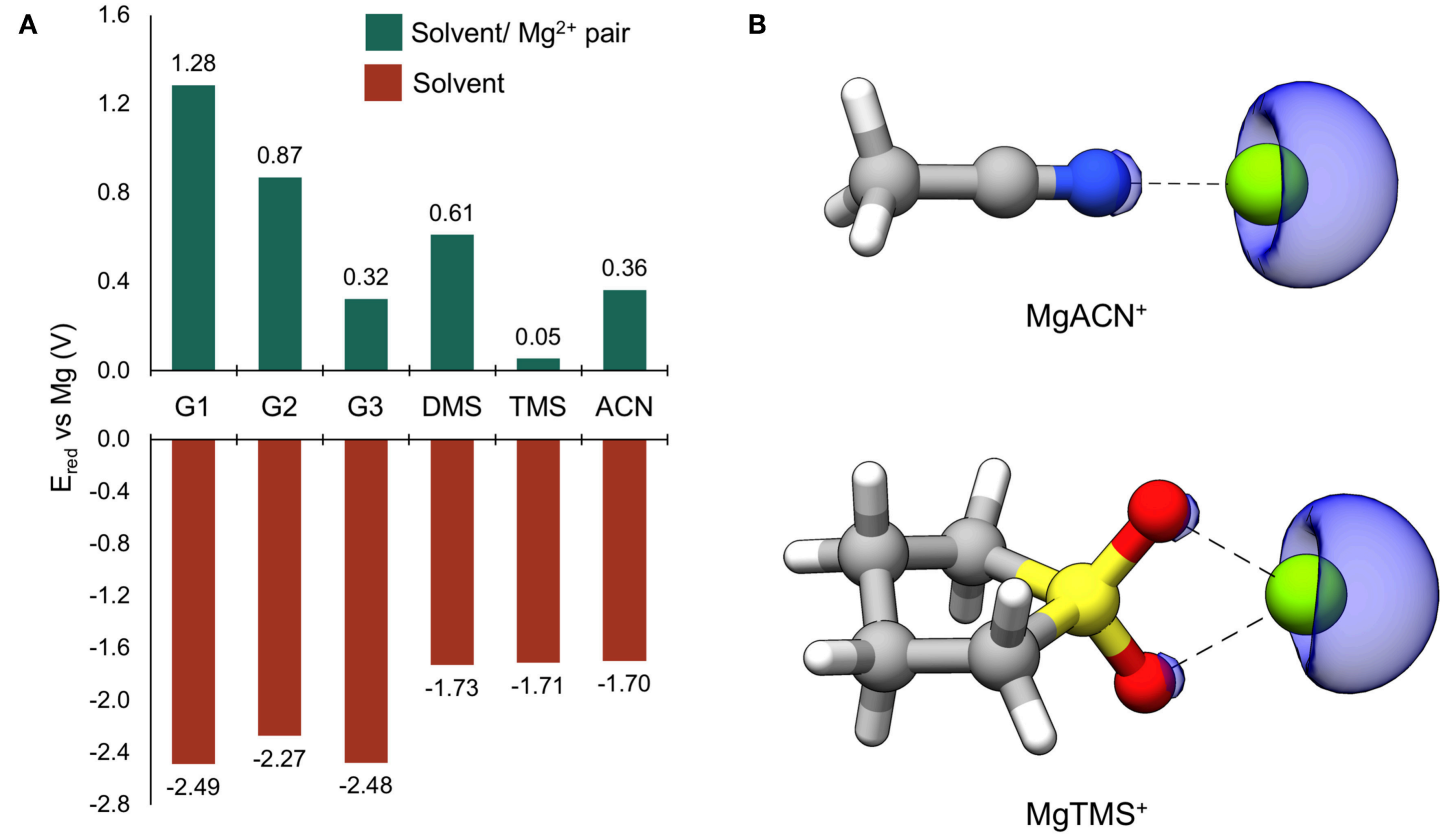
**(A)** Computed reduction potentials vs. Mg (V) for individual solvent molecules (red bars) and coordinated solvent/Mg^2+^ pairs (green bars). **(B)** The ACN/ and TMS/Mg^2+^ dimers following reduction. The blue surface is the spin density at an isosurface value of 0.006 au.

Reduction potentials were computed for solvents considered in this study as monomers and when coordinated to Mg^2+^. The results are given in Figure 2A. All reduction potentials for the uncoordinated solvents were found to be negative, indicating intrinsic stability against reduction at Mg metal potentials. The values for the glymes are the lowest and below −2.0 eV vs. Mg, followed by those of DMS, TMS, and ACN which are significantly higher (−1.73, −1.71, and −1.70 V vs. Mg, respectively), though still substantially negative. This prediction of intrinsic solvent stability with respect to reduction by Mg^0^ suggests that any reductive instability must be mediated by other processes; such as explicit surface-absorption enhanced reduction or reduction through interaction with partially reduced species formed during cycling. Indeed, the values of E_red_ vs. Mg for all Mg^2+^-coordinated solvents are positive, indicating that reduction of these bound pairs is expected at potentials positive of Mg^0^. This dramatic shift is likely due to the site of reduction changing to the coordinated Mg^2+^ ion (Rajput et al., 2015). To confirm, spin density maps were obtained for the reduced ACN/Mg^2+^ and TMS/Mg^2+^ pairs and given in Figure 2B. The regions of spin density are clearly associated with the Mg center, indicating that Mg^2+^ accepts the new electron, leading to formation of partially reduced, transient solvent/Mg^+^ pairs. These results show that solvent interactions with the Mg^2+/+^ cation are critical to controlling their parasitic reduction and must be considered in the design of electrolytes for Mg batteries.

Through investigating the dependence of the computed reduction potentials on the van der Waals scaling factor and degree of explicit Mg^2+^ coordination, it was found that the computed reduction potential for a solvent molecule is relatively insensitive to the scaling factor, while the computed reduction potential of Mg^2+^ is highly dependent on the scaling factor as well as the degree of explicit solvent coordination. Hence, the value of E_red_ should be taken as a qualitative guide; however, in all cases, the cation remains the site of reduction and Mg^+^ species are formed upon reduction of the coordinated Mg^2+^ species. See the Supplementary Material for more details.

### Mg^+^-Mediated Solvent Decomposition

As alluded to in the introduction, ideal Mg electrolytes are assumed to favor dissociated salts, rendering the coordination environment of Mg^2+^ cations as composed of solvent. The burden of chemical stability during plating would thus fall on the solvent of the electrolyte. To assess chemical stability during these conditions, decomposition reaction profiles were explored for solvents coordinated to Mg^+^. Electrolyte stability is examined under bulk electrolyte conditions, i.e., in the absence of any explicit surface interactions. The solvent molecule undergoing decomposition was treated as part of a full, explicitly modeled solvation shell coordinated to Mg^+^, representing the species present after the first electron transfer step toward Mg plating. The effect of solvation beyond this shell is accounted for with the PCM method (see section Theoretical Methods).

**Figure 3:**
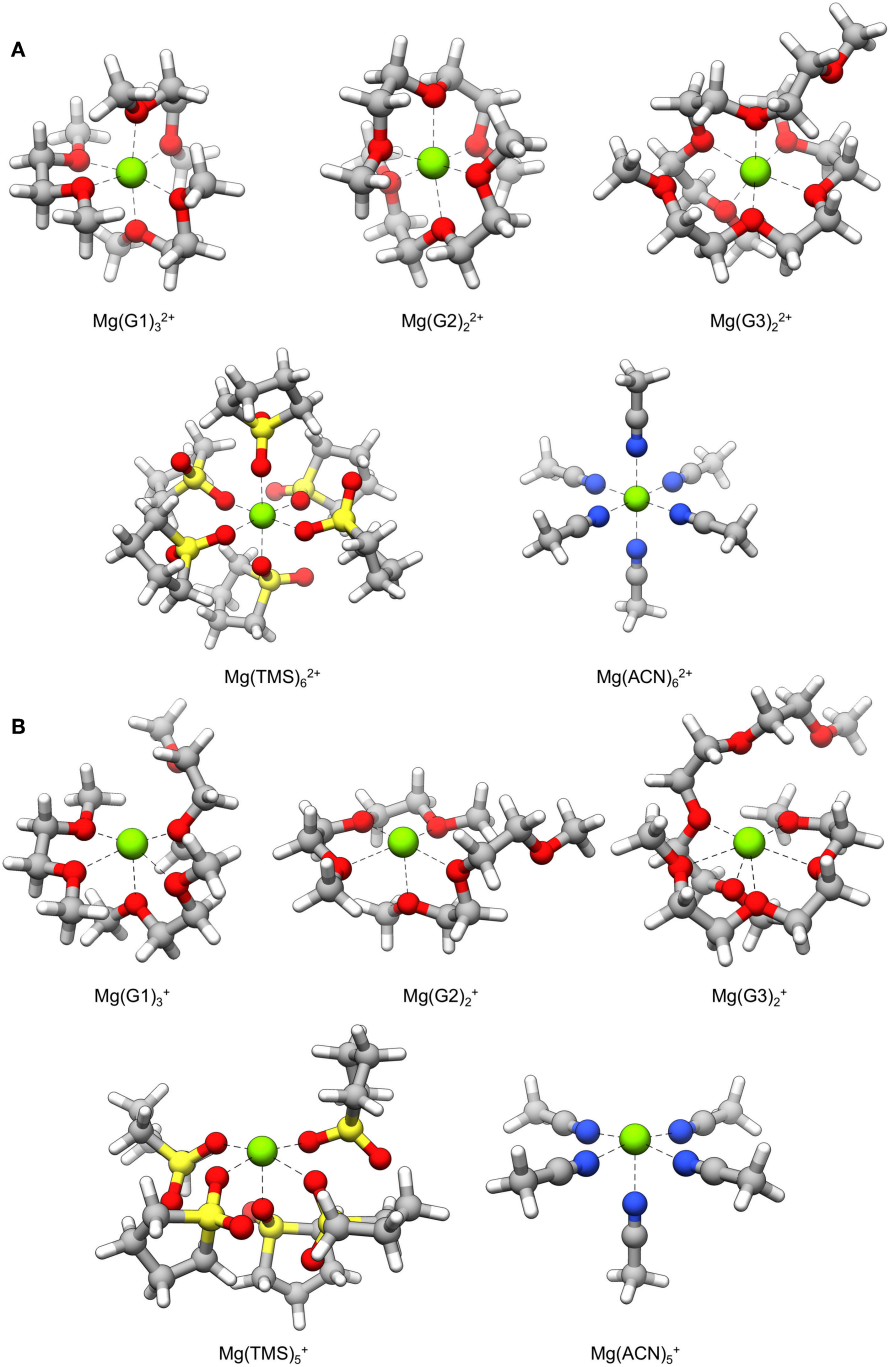
Solvent coordination complexes containing a **(A)** Mg^2+^ and **(B)** Mg^+^ center. The Mg^+^ complexes are used as starting points for the decomposition mechanisms explored in this study.

Mg^+^ coordination complexes used for decomposition reaction pathways are shown in Figure 3B. In most cases, Mg^+^ exhibits a coordination number of five with the ligated atoms arranged in a square pyramidal geometry; the exception is the $\text{Mg}{\left(\text{G}3\right)}_{2}^{+}$ complex where the coordination number is six and the geometry is pentagonal pyramidal. In all complexes, there is an open coordination site due to occupation of an unpaired electron on Mg^+^. The Mg^2+^ versions of these complexes are shown in Figure 3A for comparison; these complexes show the hexacoordinate binding environment typically seen of Mg^2+^ ions. These structures reveal that loss of a coordinating solvent atom is favored after reduction of Mg^2+^ to Mg^+^, and that octahedral symmetry may no longer preserved during this transient state. However, we note that the life-time of the Mg^+^ will compete with the residence time of solvent rearrangement.

**Figure 4:**
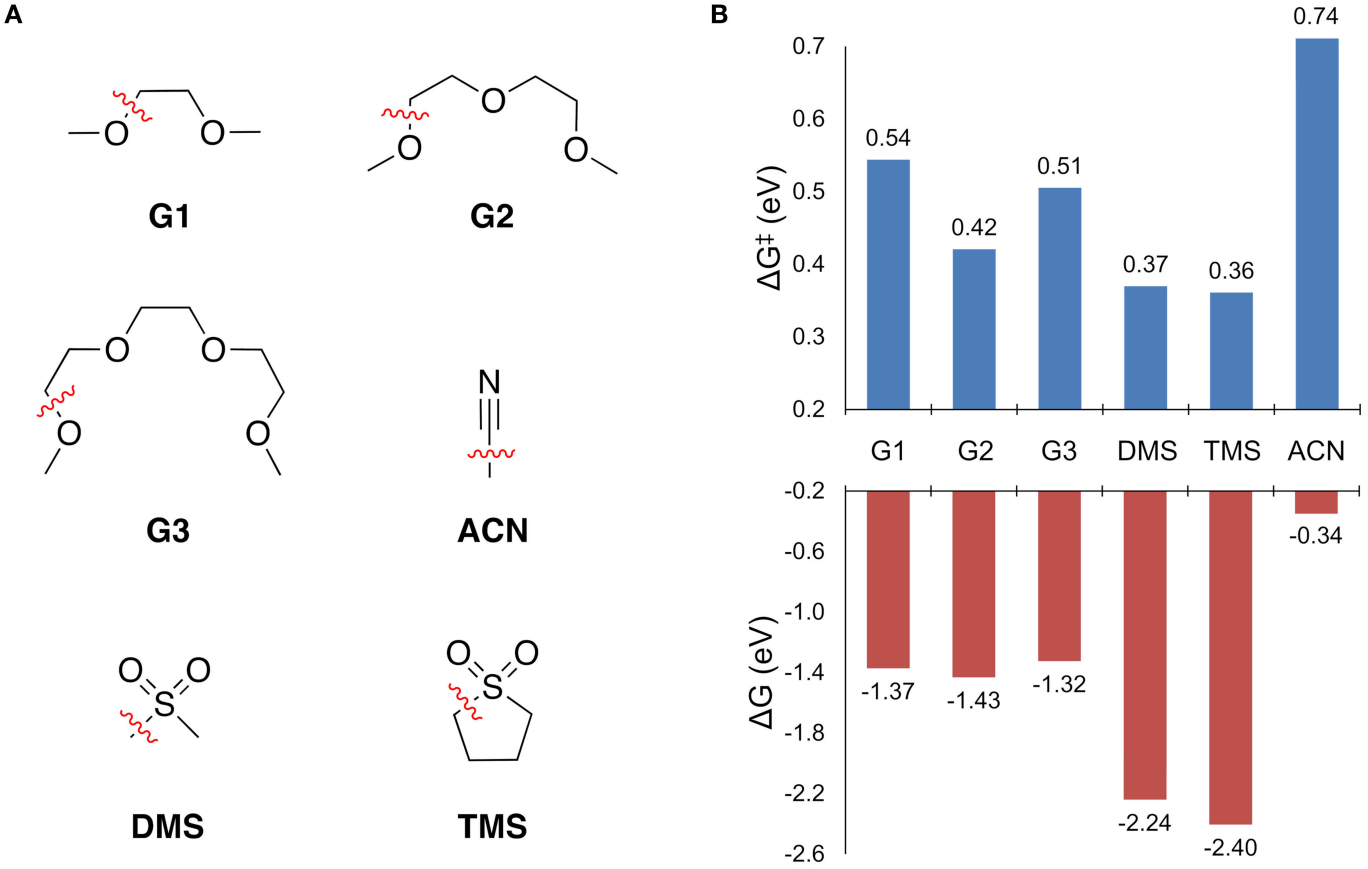
**(A)** The solvents considered for this study and the operative decomposition pathway while coordinated to Mg^+^ as indicated by the dashed (broken) bond. **(B)** The free energy change to break the indicated bond (ΔG), and the associated free energy barrier height (ΔG^‡^).

Solvent stability was first assessed by screening the free energy change to break all of the unique bonds of a solvent molecule in the solvation shell. For bond dissociations that were exergonic, the decomposition reaction profile was obtained to find the bond dissociation with the lowest barrier height. The results are shown in Figure 4. The free energy change to break all other bonds is given in the Supplementary Material.

Every solvent in Figure 4B shows an exergonic bond dissociation, which taken alone might suggest that none are suitable for Mg electrolytes. However, the widespread success of the glymes in achieving high efficiency Mg plating is unquestionable. Therefore, complete reaction profiles for these bond dissociation events were computed to determine whether the kinetic barrier to decomposition is another necessary factor in deciding the stability of the solvents in Figure 4. From Figure 4B, the sulfones exhibit the lowest barriers (0.36 and 0.37 eV for TMS and DMS, respectively), followed by the glymes (ranging from 0.42 to 0.54 eV for G2 and G1, respectively), and then finally ACN (0.74 eV). The predicted stability of ACN toward bond dissociation is quite surprising given its lack of demonstrated ability to support efficient Mg plating and stripping. However, this discrepancy is explained by the fact that ACN shows the *lowest barrier* for Mg^+^ to solvent electron transfer (*vide infra*). Reaction profiles for selected solvents are discussed in detail in the following sections.

### Diglyme

**Figure 5:**
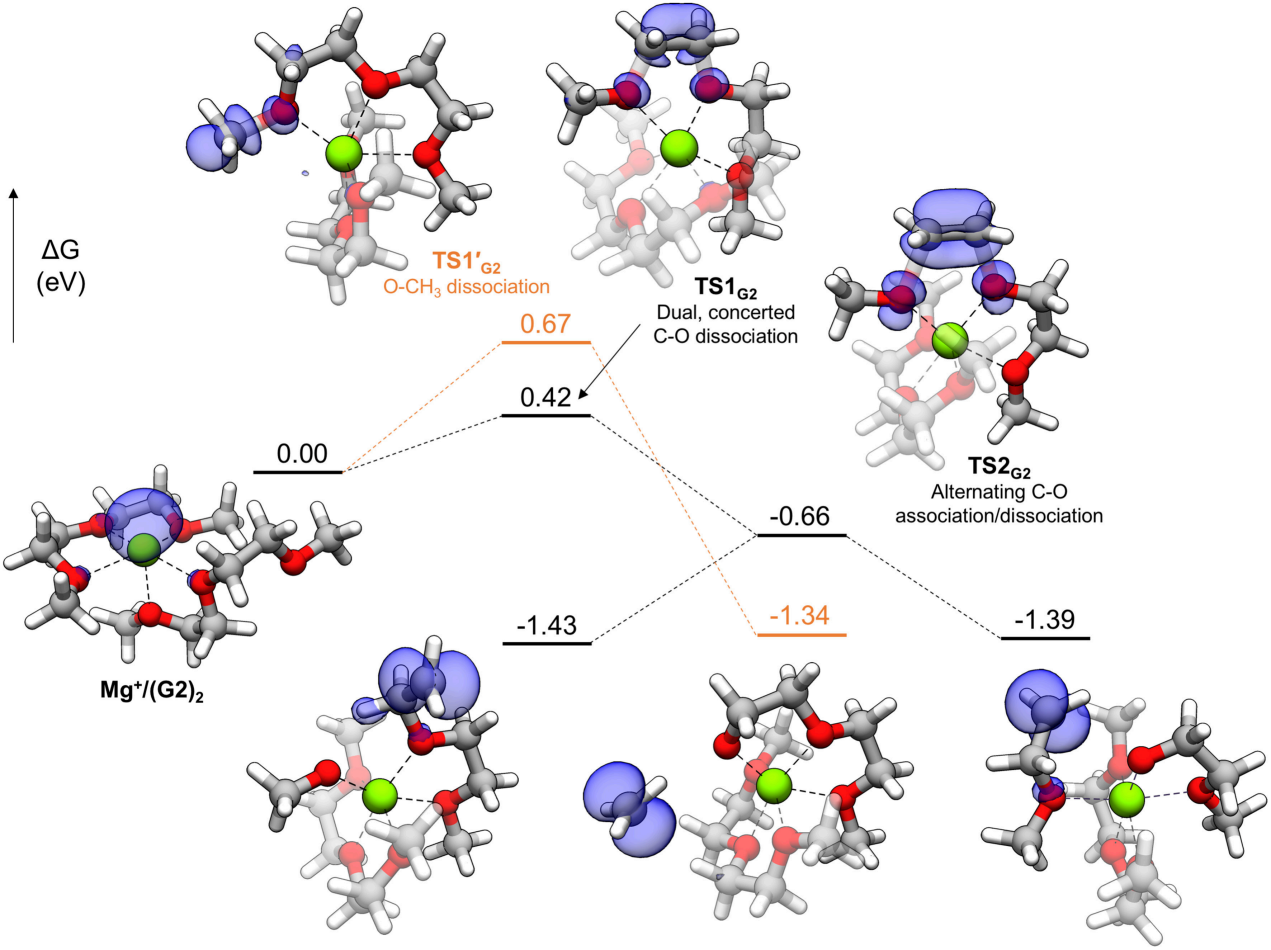
Decomposition reaction profile for the $\text{Mg}{\left(\text{G}2\right)}_{2}^{+}$ complex. The lowest barrier pathway to dissociate a non-terminal C-O bond is the black line; the orange line is an alternate pathway to dissociate a methyl radical. The blue regions correspond to electron spin density at an isosurface value of 0.006 au.

**Figure 6:**
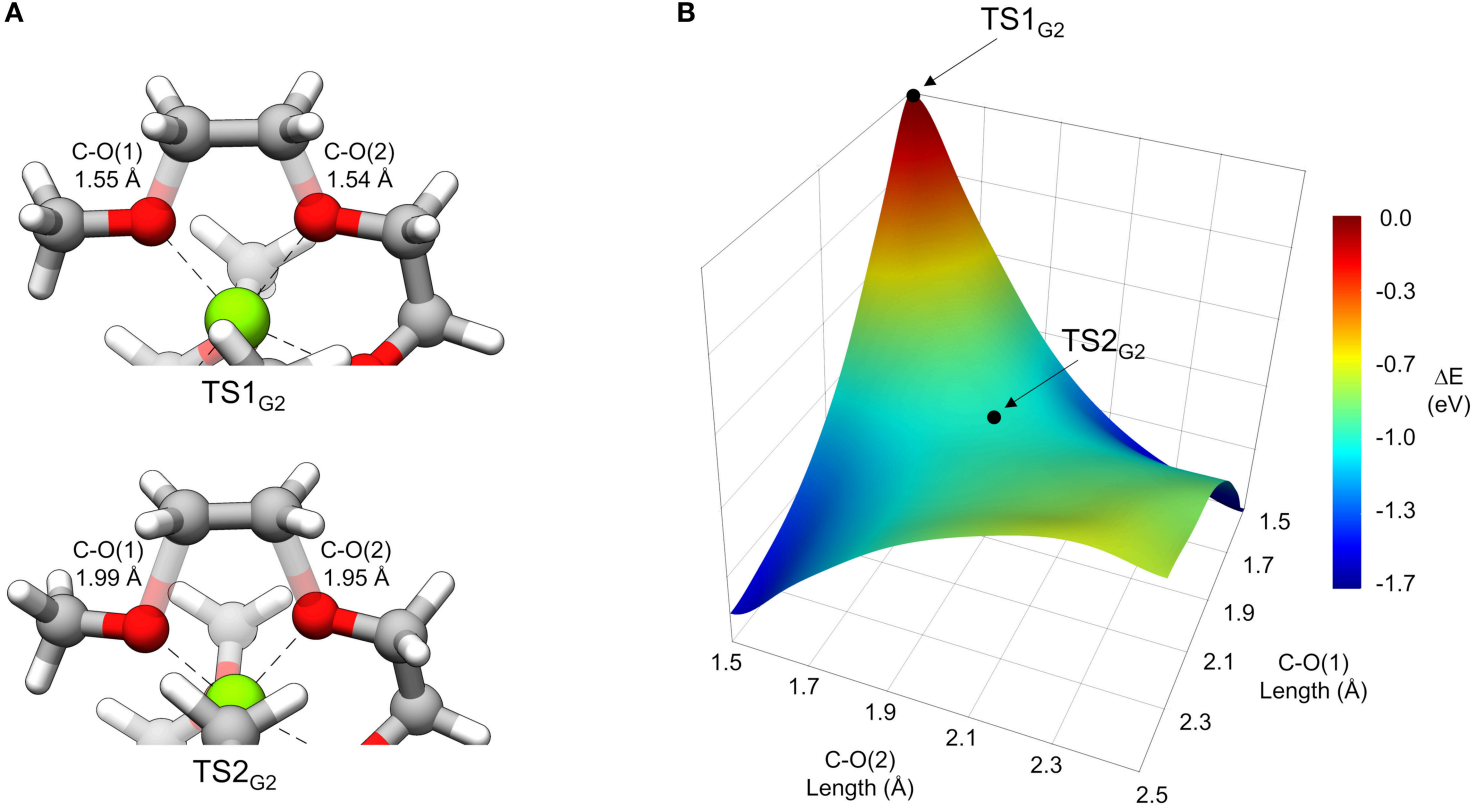
**(A)** A view of **TS1**_G2_ and **TS2**_G2_ that more closely shows the difference in C-O transition bond lengths. **(B)** 3-dimensional contour plot of energy (eV) relative to **TS1**_G2_ at the PCM/wB97X-D/6-31g(d) level of theory as a function of the two C-O bond lengths from 1.5 to 2.5 Å.

In general, thermodynamically favorable bond dissociations for the glymes take place at the C-O bonds. We found, in exploring different reaction mechanisms, that the rate only depends on whether the dissociating C-O bond is terminal or non-terminal. These two pathways are shown for diglyme in Figure 5. The spin density (indicated by the blue region) shows that the initial structure contains the unpaired electron on the Mg^+^ center and allows tracking of this electron as the reaction proceeds. The lowest energy configuration for the starting complex is pentacoordinate, with one of the six solvent oxygens (from two molecules) remaining uncoordinated. The barrier to dissociate a terminal methyl radical, represented by **TS1′**_G2_, is 0.67 eV, and this reaction leads to the products in a single step. The barrier to dissociate a non-terminal C-O bond is 0.42 eV, given by **TS1**_G2_, and this was the lowest energy barrier located that would initiate any sort of decomposition process. **TS1**_G2_ actually shows what appears to be dissociation of an ethylene radical cation by nearly equal elongation of two adjacent C-O bonds. Also, notably, the unpaired electron has already moved to this fragment in the transition state, re-oxidizing the metal center to Mg^2+^ and restoring a hexacoordinate configuration by adopting coordination with the oxygen that was previously uncoordinated in the starting complex. An intrinsic reaction coordinate (IRC) calculation for **TS1**_G2_ showed dissociation of the ethyl radical fragment early in the reaction coordinate, which reaches a limited degree of displacement before spontaneously reforming one of the two initial C-O bonds (see Supplementary Material). The only stationary point between **TS1**_G2_ and the decomposed products is **TS2**_G2_, in which the two transition bond lengths are more elongated and reflect alternating C-O bond formation and dissociation in the imaginary frequency, serving as a pathway for interconversion of the two product minima. Because this decomposition pathway, initiated by **TS1**_G2_, leads to two possible product configurations without an intermediate, it is an example of a potential energy surface bifurcation (Ess et al., 2008; Hare and Tantillo, 2017). To confirm, a 3-D potential energy surface was generated from constrained optimizations at fixed bond lengths for the two adjacent C-O bonds ranging from 1.5 A (approximately the length for each C-O bond in **TS1**_G2_), to 2.5 A. The surface is shown as a contour plot in Figure 6 together with a closer look at the two bonds in **TS1**_G2_ and **TS2**_G2_.

The same pathway was identified as the lowest energy decomposition process for G1 and G3, involving a transition state with equal elongation of adjacent non-terminal C-O bonds and leading to two possible products where one bond reforms and the other breaks. Despite this similarity in the transition state for each of the glymes, the barrier against decomposition varies slightly and follows the order G2<G3<G1, ranging from 0.42 to 0.54 eV. Because of the small variation in barrier height, a difference in stability among the glymes may not be readily observable from these considerations. While diglyme has been preferred in many recent studies, to our knowledge, there is no conclusive evidence of differing plating stability among the glyme family. Typically, the choice of glymes in Mg electrolytes is dictated by salt dissociation ability, impurity levels, and viscosity considerations. It should be noted that longer chain length glymes have been associated with better solubility of Mg salts (Rajput et al., 2015). This has practical consequences on electrolyte stability by varying the coordination environment of Mg^2+^ cations and stability against Mg^+^-mediated reductive and/or decomposition processes.

To probe the origins of the differences in stability of the glymes, a distortion/interaction analysis (Ess and Houk, 2007) is applied, with terms accounting for the oxidation and reduction that occurs between the starting complex and the transition state:

$$\begin{array}{l} \Delta {\text{E}}^{‡}=\Delta {\text{E}}_{\text{dist}}\left(\text{glyme}\right)+\Delta {\text{E}}_{\text{int}}+\Delta {\text{E}}_{\text{red}}\left(\text{glyme}\right)+\;\Delta {\text{E}}_{\text{ox}}\left(\text{M}{\text{g}}^{+}\right), \end{array}$$

where ΔE^‡^ is the reaction barrier height; ΔE_dist_(glyme) is the difference in energy of the glymes between their transition state and reactant geometries; ΔE_int_ is the difference in interaction energy between the transition state and reactant complexes, where the interaction energy is the energy of the intact complex minus the energy of the species in the complex as isolated fragments; ΔE_red_(glyme) is approximately the energy to reduce the glyme molecule undergoing decomposition; and ΔE_ox_(Mg^+^) is the energy to oxidize Mg^+^. ΔE_red_(glyme) was found from the difference in energy of the decomposing glyme molecule with a negative charge and with no charge. The terms for oxidation and reduction is included in this analysis by noting that oxidation of Mg^+^ and reduction of a glyme molecule has already taken place by the transition state in the reaction coordinate for glyme decomposition, as discussed above for the diglyme case. All energies in this analysis were performed in the gas phase to exclude effects of entropy and implicit solvation by the PCM method. These energies together with the solution phase free energy barriers for comparison are shown in Table 1.

**Table 1:** Solution-phase free energy barrier (ΔG^‡^), gas-phase energy barrier (ΔE^‡^), difference in energy of the glymes between the transition state and reactant complex geometries [ΔE_dist_(glyme)], reduction energy of the glymes [ΔE_red_(glyme)], difference in interaction energy between the transition state and reactant complex geometries (ΔE_int_), and oxidation energy of Mg^+^ [ΔE_ox_(Mg^+^)] for the decomposition reactions of G1, G2, and G3, in eV.

**Solvent**	**ΔG^**‡**^**	**ΔE^**‡**^**	**ΔE_dist_(glyme)**	**ΔE_red_(glyme)**	**ΔE_int_**	**ΔE_ox_(Mg^**+**^)**
G1	0.54	0.68	0.91	1.63	−16.56	14.70
G2	0.42	0.55	0.98	1.68	−16.81	14.70
G3	0.51	0.58	0.77	1.56	−16.44	14.70

**Figure 7:**
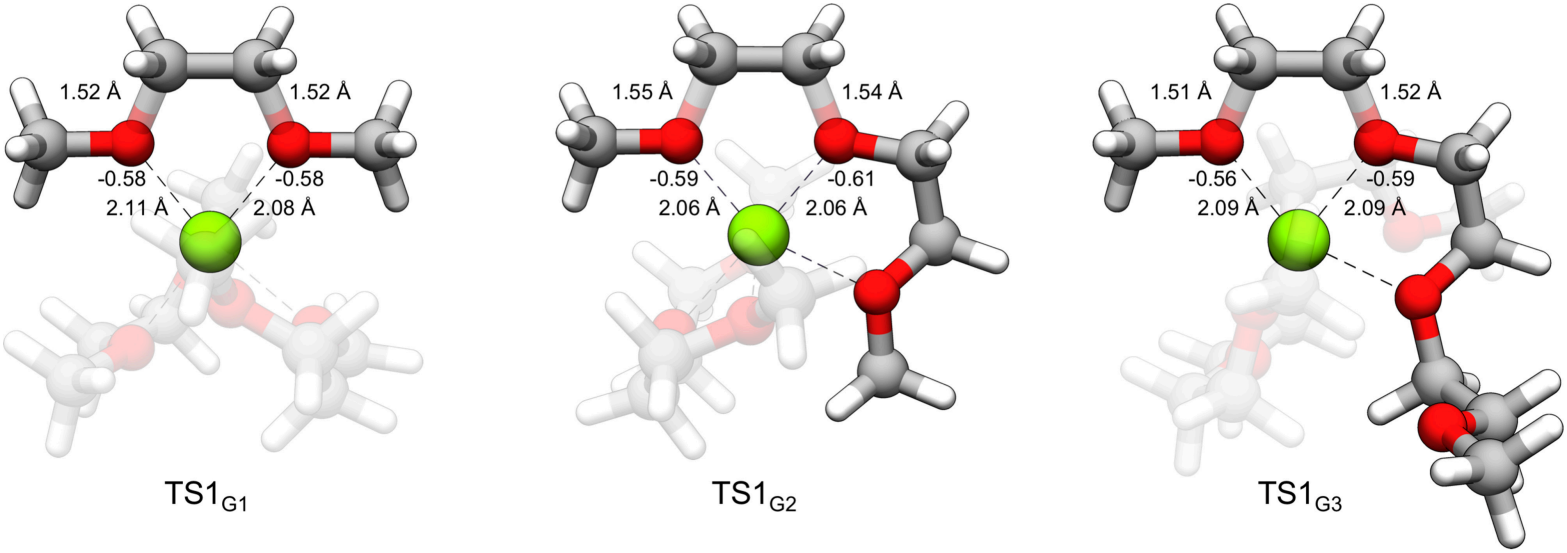
**TS1**_G1_, **TS1**_G2_, and **TS1**_G3_ with labeled C-O bond lengths, O···Mg^2+^ interaction distances and natural atomic charges critical to the distortion and interaction energies comprising the difference in decomposition barrier heights. The charges for oxygen were obtained for the geometry of the glymes of the complex in the absence of Mg^2+^ and assigned an overall charge of –1.

From Table 1, while the solution phase ΔG^‡^ and gas-phase ΔE^‡^ values correlate, the ΔE^‡^ values are all slightly larger than the ΔG^‡^ values, indicating that longer range solvation, and entropy contributes to a lowering of the activation barrier. The value for ΔE_ox_(Mg^+^) is 14.70 eV throughout. The strongly negative ΔE_int_ values reflect formation of hexacoordinate Mg^2+^ complexes in the transition state from the pentacoordinate Mg+ starting complexes and should be considered dependent on the time-scale of the two processes, as mentioned previously. The ΔE_int_ and ΔE_ox_(Mg^+^) values are the largest contributors to ΔE^‡^ in all cases. The ΔE_red_(glyme) values vary slightly around ~1.6 eV. The data in Table 1 show, intuitively, a correlation between ΔE_dist_(glyme) or ΔE_red_(glyme) and ΔE_int_ in which an increase in energy of the glymes accompanies a more negative difference in interaction energy. Figure 7 shows each of the transition state structures with labels for the C-O bonds undergoing association/dissociation, interaction distances between Mg^2+^ and the oxygens of these bonds, and these oxygens' computed natural atomic charges.

From Table 1 and Figure 7, it could be noted that ΔE_dist_(glyme) is related to the extent of C-O bond elongation in the transition state. These bonds are slightly longer in **TS1**_G2_ than the other transition states (see Figure 7). The G2 decomposition reaction has the highest ΔE_dist_(glyme) (0.98 vs. 0.91 and 0.77 eV for G1 and G3, respectively). At the same time, the oxygens in the C-O bonds undergoing association/dissociation bear a slightly more negative charge, and the O···Mg^2+^ interaction distances are shorter (see Figure 7). Thus, the slightly lower barrier to decomposition of G2 vs. the other glymes could be seen as resulting from a greater extent of C-O bond dissociation, itself a destabilizing effect, overcompensated for with stronger interactions between the cation and the oxygens which have more buildup of negative charge.

### TMS

**Figure 8:**
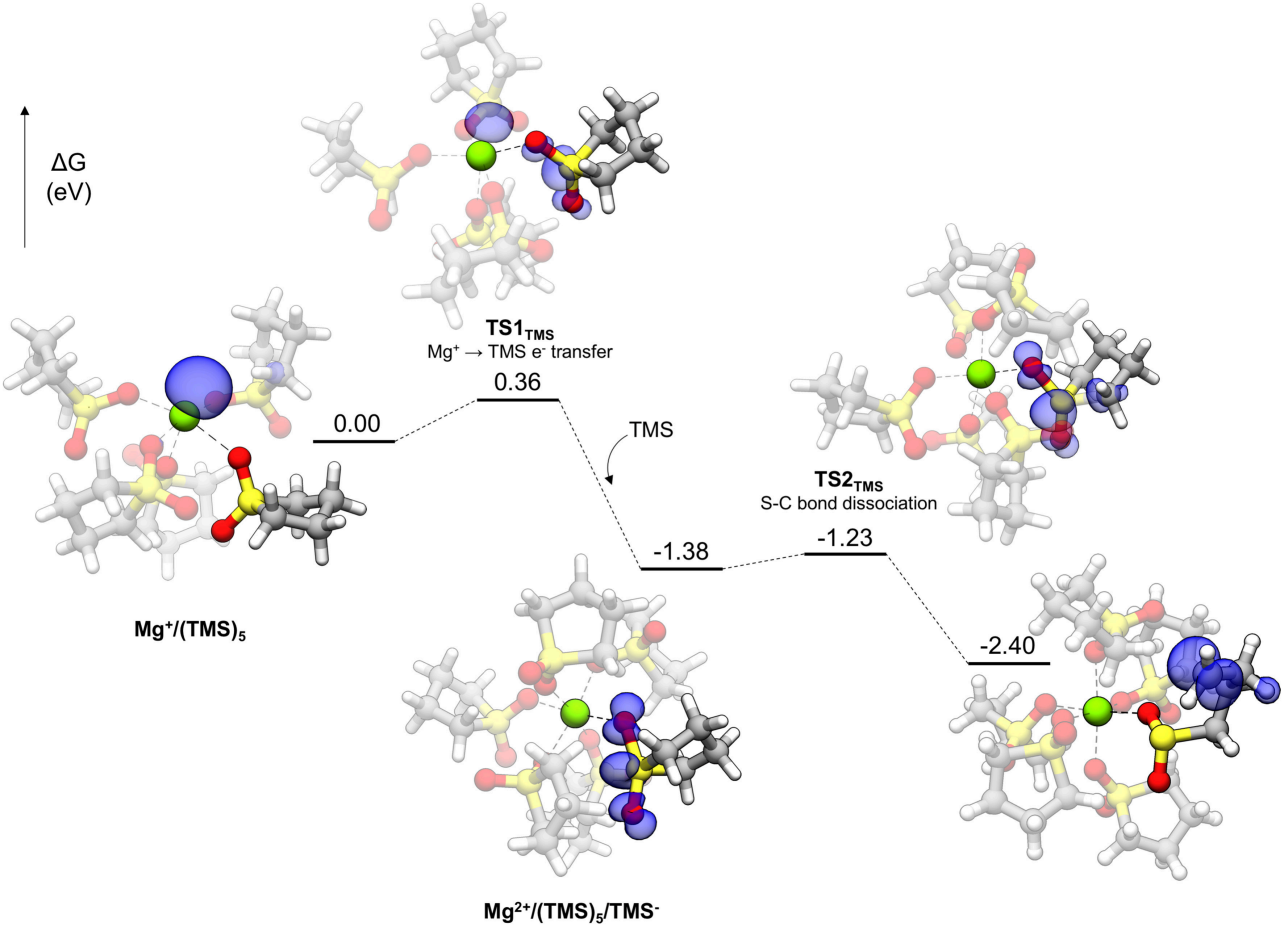
Decomposition reaction profile of TMS starting from a $\text{Mg}{\left(\text{TMS}\right)}_{5}^{+}$ complex. The blue regions correspond to electron spin density at an isosurface value of 0.006 au.

Polar solvents like TMS are interesting for Mg energy storage applications for their relatively high anodic stability and solvating ability if not for their poor electrolyte performance (*vide infra*). The decomposition profile for TMS is shown in Figure 8. Initially, the Mg^+^ cation is pentacoordinated to five TMS molecules. Each TMS molecule binds to Mg^+^ with one oxygen, with the other bound to a nearby TMS molecule in a CH···O interaction, comprising a network of such interactions among the TMS molecules. The decomposition of TMS begins by proceeding to **TS1**_TMS_, in which the unpaired electron of Mg^+^ begins transferring to the solvent. The spin density of **TS1**_TMS_ shows that this unpaired electron is shared between both the cation and the TMS molecule. After electron transfer, the unpaired electron is delocalized across the sulfonyl group and a notable geometric change is the widening of the O-S-O angle (from 116.1° initially to 131.5° in **TS1**_TMS_ and then 154.7° in the following intermediate) due to accommodation of the unpaired electron. After the electron transfer, the Mg cation becomes Mg^2+^ and hexacoordinate by adopting another TMS molecule in the coordination shell [**Mg**^**2+**^**/(TMS)**_5_**/TMS**^**−**^ in Figure 8]. The hexacoordinate version of this complex is lower in energy than the pentacoordinate plus a dissociated TMS molecule by 0.60 eV. Finally, a C-S bond dissociates via **TS2**_TMS_ with a small barrier of 0.15 eV. The overall barrier to decomposition for TMS is associated with **TS1**_TMS_ (0.36 eV).

DMS, with a very similar barrier to decomposition, is a smaller molecule than TMS containing the sulfonyl functionality and is actually a solid far above room temperature (the melting point is 110°C). It was used as a model for exploring sulfonyl decomposition pathways prior to obtaining the analogous TMS versions. The pathway for DMS is the same as TMS, with a very similar overall barrier, as given in Figure 4B (0.37 eV). The barrier for the second step in the pathway is 0.21 eV. The overall barrier for other sulfonyl-based solvents (e.g., 3-methyl sulfolane) is expected to be similar.

### Experimental Comparison of Glyme and Sulfone Stability

**Figure 9:**
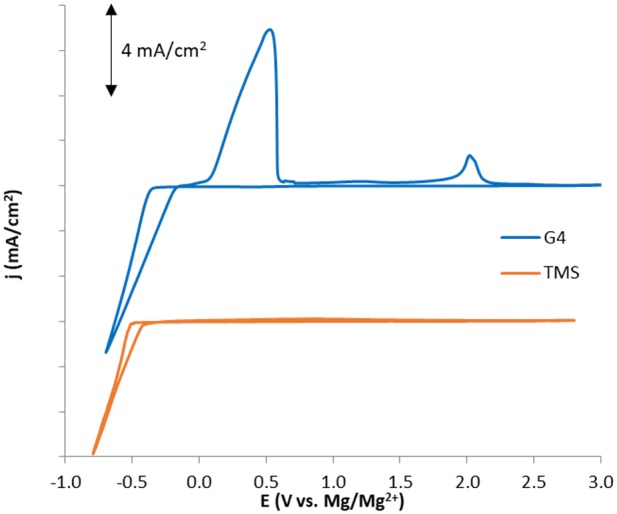
Cyclic voltammograms for 0.5 M MgTFSI_2_ solutions in G4 and TMS illustrating significant differences in Coulombic efficiency consistent with computed differences in solvent stability during Mg plating/stripping.

To experimentally compare the reductive stabilities of glyme and sulfone-based solvents under similar conditions, cyclic voltammograms (CVs) of Mg plating and stripping were obtained for purified electrolyte mixtures of 0.5 M Mg(TFSI)_2_ dissolved in G4 and TMS (Figure 9)^1^. Although reduction current is attained at similar rates and with only slightly different onset potentials in both solvents, the Coulombic efficiency is much lower in TMS than in G4 (<20 vs. 78%), indicating a significantly greater rate of parasitic reduction accompanying Mg^2+^ reduction in the sulfone. A similar degree of irreversibility was observed in work by Senoh et al. which examined the electrochemical behavior of MgTFSI_2_ solutions in a few sulfone solvents, including TMS. While X-ray diffraction data showed that Mg plating was possible in this electrolyte, the extent of Mg plating achieved relative to other parasitic processes was not certain (Senoh et al., 2014).

**Figure 10:**
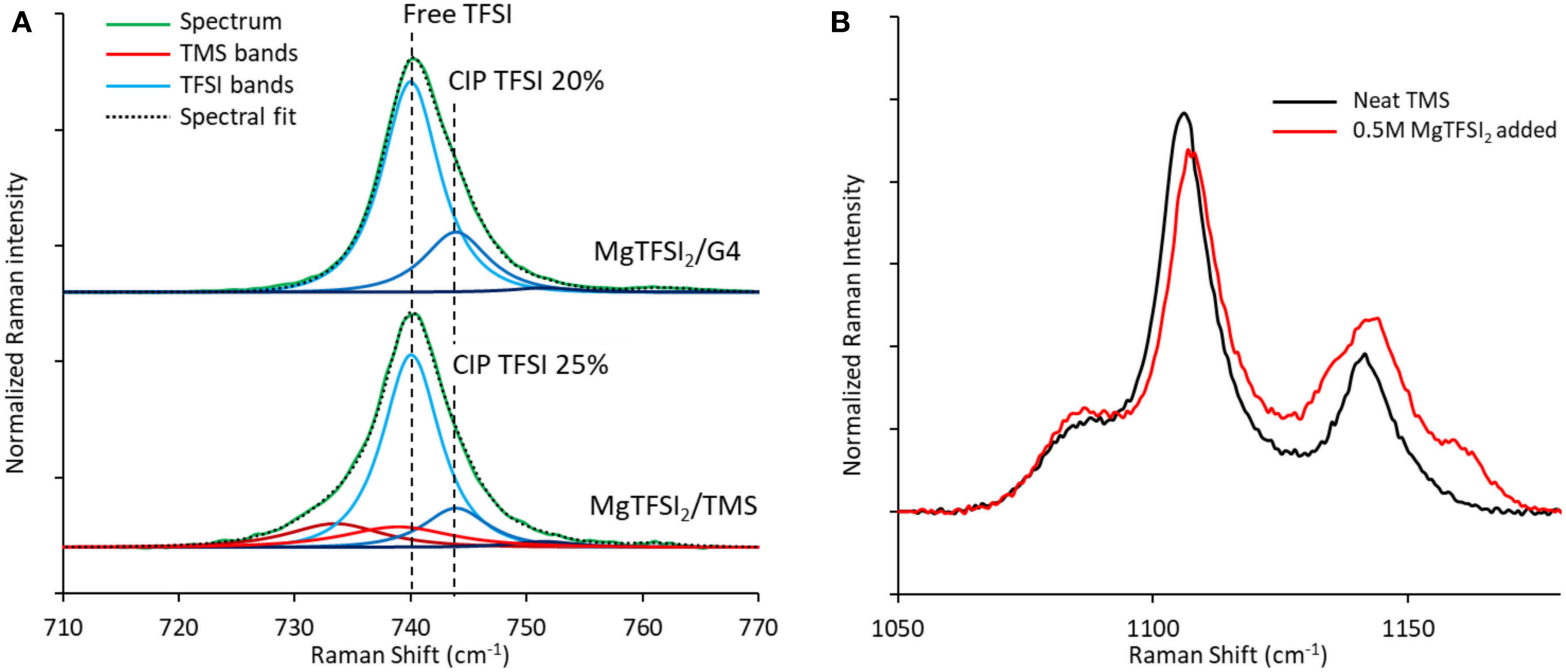
Raman spectra of TFSI electrolyte formulations. **(A)** Comparison of the Raman bands of the TFSI anion show similar extents of ion pairing for 0.5 M MgTFSI_2_ dissolved in either G4 or TMS. **(B)** TMS Raman bands in the *v*(SO_2_) region confirm strong interactions between TMS and Mg^2+^ after MgTFSI_2_ salt addition.

Comparison of the TFSI breathing mode in Raman spectra generated for these two solutions demonstrates a similar fraction of contact-ion-paired TFSI of 21–25% (Figure 10A), arguing that the loss of Coulombic efficiency moving from G4 to TMS is governed by the stability of the solvent rather than by differences in TFSI reduction resulting from ion pairing (Rajput et al., 2015). Consistent with the formation of a significant free TFSI population, a Raman spectral comparison of neat TMS and a 0.5 M MgTFSI_2_ solution in TMS confirms strong solvent interaction with Mg^2+^ based on the blue shifting and broadening of the *v*(SO_2_) band at 1,105 cm^−1^ (Figure 10B) (Legrand et al., 1996). The combination of strong TMS:Mg^2+^ interactions coupled with lower measured Coulombic efficiency supports the prediction that sulfone solvents are less stable than glyme solvents during Mg plating due to their lower barrier for decomposition when coupled to Mg^+^. However, while the barriers for decomposition of the sulfones are lower than those of the glymes, they are still significantly large, indicating sulfone stability should be attainable if the relevant electrolyte interactions are controlled. To our knowledge, the only documented examples of high Mg plating Coulombic efficiency (>90%) in sulfone solvents have utilized MgCl_2_ as the primary salt and in most cases require an ether co-solvent (Kang et al., 2017; Merrill and Schaefer, 2018; Nakayama et al., 2018). Because of the significant Mg^2+^ complexation and interfacial activation tendencies of the Cl^−^ anion (Shterenberg et al., 2015, 2017), these examples suggest that stabilization of a sulfone solvent is possible if the electrolyte composition is rationally designed such that the solvent is less exposed to the Mg^+^ cation.

### ACN

**Figure 11:**
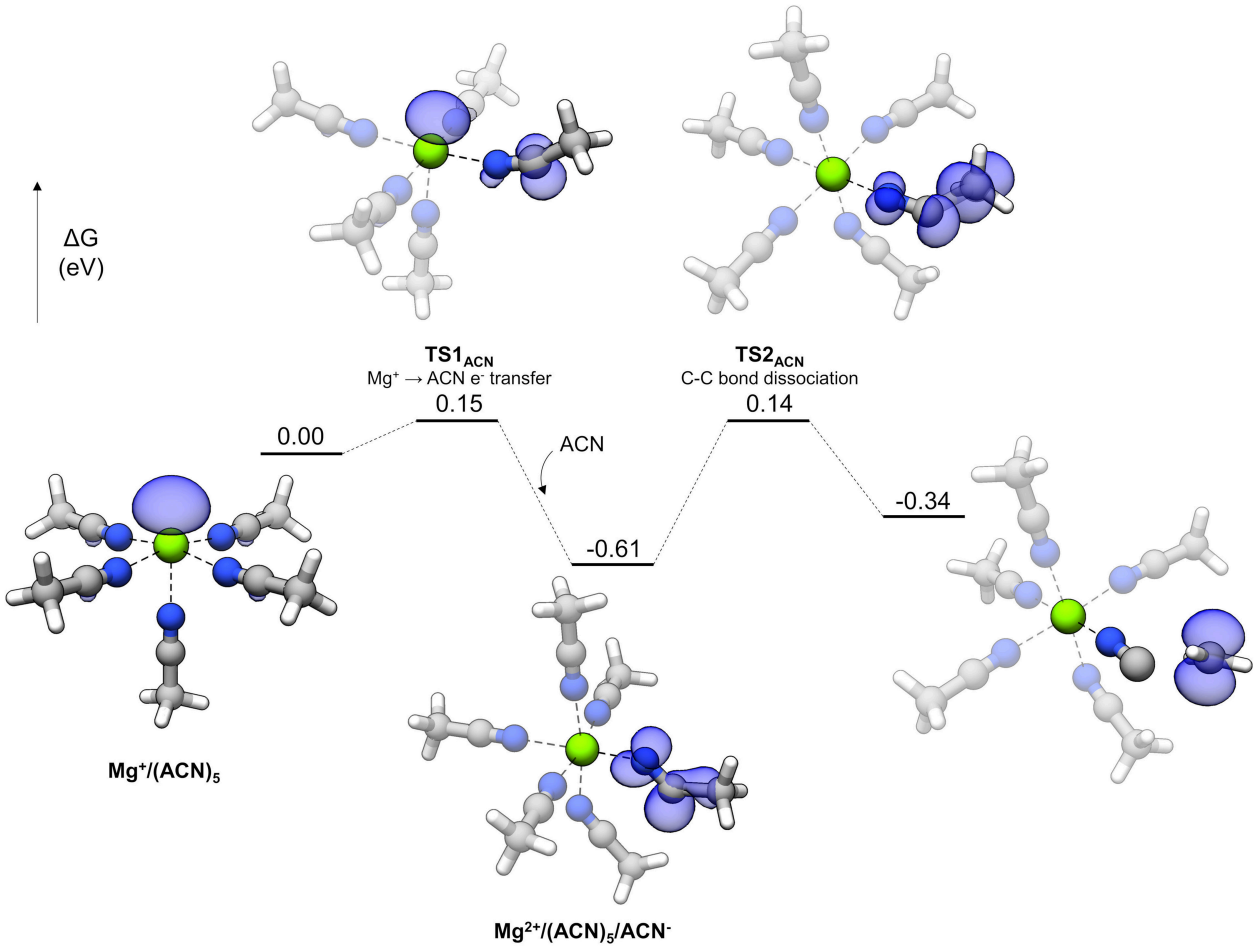
Decomposition reaction profile of ACN starting from a $\text{Mg}{\left(\text{ACN}\right)}_{5}^{+}$ complex. The blue regions correspond to electron spin density at an isosurface value of 0.006 au.

The reaction profile for ACN decomposition is given in Figure 11. The overall mechanism is similar to the sulfones' and involves charge transfer and bond dissociation as two separate steps. The starting complex is a pentacoordinate Mg^+^ complex with five coordinated ACN molecules. **TS1**_ACN_ represents the initial charge transfer step with a very small barrier of 0.15 eV. The electron spin density shows sharing of the unpaired electron between the Mg cation and an ACN molecule, which is similar to the initial step of sulfone decomposition. However, the lower barrier in the case of ACN indicates that reduction of this solvent is significantly more favorable. After **TS1**_ACN_, the reduced ACN molecule distorts further to host the unpaired electron (the N-C-C angle changes from nearly linear initially to 159.5° in **TS1**_ACN_ and then 128.8° as the reduced ACN^−^ intermediate), and the Mg ion is oxidized to Mg^2+^, allowing another ACN molecule to bind and render Mg^2+^ hexacoordinate (this hexacoordinate complex is lower in energy than the pentacoordinate version plus a dissociated ACN molecule by 0.14 eV). From here, a barrier of 0.74 eV via **TS2**_ACN_ must be overcome to dissociate the C-C bond. There are two peculiarities about the overall mechanism for ACN. One is that the intermediate reduced ACN species is lower in energy by 0.26 eV than the final decomposed products; also, the barriers for this intermediate both to decompose (0.74 eV) and go in the backwards direction to transfer the electron to Mg^2+^ (0.76 eV) are relatively large. What happens to the ACN^−^ radical in this “energy well” is unclear. Possible fates include reaction with other electrolyte species or adsorption to the electrode surface. However, the fast initial electron transfer process by **TS1**_ACN_ may lead to difficulties in Mg plating if the ACN^−^ radical is kinetically stable against transferring the electron back to Mg^2+^. This fast reduction by coordinated Mg^+^ could account for difficulties in Mg plating with ACN-based electrolytes.

## Conclusions

The suitability of some common solvents for non-aqueous Mg electrolytes with respect to stability during plating is examined with DFT calculations together with supportive cyclic voltammetry as well as Raman spectroscopy. All reduction potentials for the uncoordinated solvents were found to be negative, indicating intrinsic stability against reduction at Mg metal potentials. Upon reduction of solvent/Mg^2+^ coordinated pairs, reduction preferentially takes place on the cation, forming solvent/Mg^+^ pairs. All of the solvents exhibit exergonic decomposition pathways while coordinated to the transient Mg^+^ species formed upon metal deposition. Interestingly, reaction profiles for the decomposition of each solvent shows that stability varies with the kinetic barrier to decomposition, with ACN exhibiting the highest barrier, followed by the glymes, and then the sulfones. The decomposition mechanism for the glymes involves cleavage of a non-terminal C-O bond that follows from initial dissociation of an ethylene radical cation and spontaneous reformation of one C-O bond or the other via a potential energy surface bifurcation. The mechanism for the sulfones and acetonitrile is a two-step process in which bond dissociation follows from an initial electron transfer step from Mg^+^ to the coordinated solvent. For the sulfones, the initial charge transfer is the limiting step and the subsequent barrier to dissociate a C-S bond is very small. For acetonitrile, the initial charge transfer step is very fast and the reduced ACN^−^ radical gets trapped in an “energy well” with an uncertain outcome. However, the initial charge transfer to form the ACN^−^ radical may be sufficient to cause difficulties with Mg plating if the barrier to transfer the electron back to Mg^2+^ is prohibitive. Experimental CVs of Mg(TFSI)_2_/TMS mixtures show poor coulombic efficiency, and Raman spectra of these mixtures show coordinating TMS bands and diminished coordinating TFSI bands, implying that reduction of solvent coordinated to Mg^2+^ is responsible for the poor performance. Our work strongly indicates that Mg^+^-mediated reduction and/or decomposition are responsible for the difficulties in implementing ACN or sulfones as solvents for Mg electrolytes. While our current work shows agreement with experimental observations on solvent stability, we model decomposition pathways in bulk electrolyte conditions; the influence of surfaces such as the electrolyte-electrode interphase on coordination chemistry and decomposition pathways is a topic that could be further explored. Poor performance in Mg plating with polar solvents such as ACN or those based on sulfones are representative of the difficulties in moving past the ethereal solvents, such as the glymes, which are currently the most commonly used solvents for Mg electrolytes. Insight to the issues of solvents with desirable properties such as relatively high anodic stability, which are characteristic of polar solvents like sulfones and ACN, should be useful toward rational design of improved electrolytes.

## Author Contributions

TS performed the theoretical calculations. NH performed the Raman spectroscopy and cyclic voltammetry experiments. The manuscript was written with contributions from all authors.

### Conflict of Interest Statement

The authors declare that the research was conducted in the absence of any commercial or financial relationships that could be construed as a potential conflict of interest.

## Footnotes

^1^G4 was used instead of G1-3 for comparison with TMS because, among the glymes, it had the closest extents of ion-pairing with TMS in mixtures with MgTFSI_2_. The G1-G3 mixtures showed 10–15% less ion-pairing than that with G4.

## Supplementary Material

Mg^+^ complex stability screening; additional computed reduction potentials; Intrinsic Reaction Coordinate (IRC) of G2_2_Mg^+^ decomposition; effect of alpha on the potential energy surface, and location of difficult transition states; optimized cartesian coordinates.
