# Supplementary material for: Elucidating Non-aqueous Solvent Stability and Associated Decomposition Mechanisms for Mg Energy Storage Applications From First-Principles
Source: Front Chem. 2019 Apr 9;7:175. doi: 10.3389/fchem.2019.00175 (PMC6465547; doi:10.3389/fchem.2019.00175)
Supplement: Supplementary file 1 [file Table_1.docx]

# *Supplementary Material*

Elucidating Non-aqueous Solvent Stability and Associated Decomposition Mechanisms for Mg Energy Storage Applications from First-Principles

**Trevor J. Seguin, Nathan T. Hahn, Kevin R. Zavadil, and Kristin A. Persson^*^**

*** Correspondence:** Kristin A. Persson: kristinpersson@berkeley.edu

# Mg^+^ complex stability screening


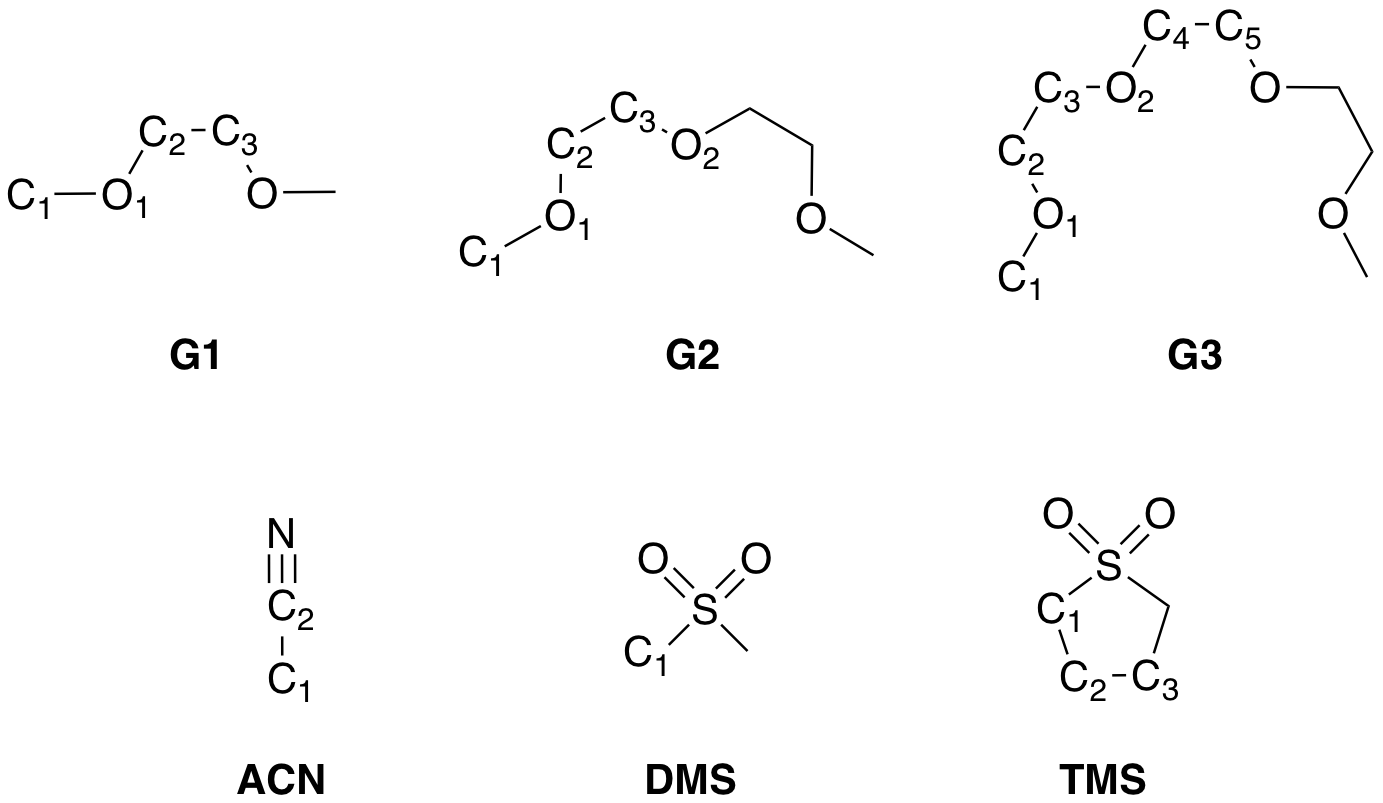


**Supplementary Figure 1**. Solvent molecules with labelled atom numbers.

**Supplementary Table 1**. Bond dissociation free energies (eV) for solvent molecules in the corresponding pentacoordinate Mg^+^ complex at the PCM/wB97X-D/6-31g(d) level of theory. For TMS, DMS, and ACN, the final state involves a hexacoordinate Mg^2+^ complex (with six solvent molecules, where one has a dissociated bond) and the energy is referenced against the energy of an initial pentacoordinate (five solvent molecule) Mg^+^ complex plus a dissociated solvent molecule.

|  | C_1_-O_1_ | O_1_-C_2_ | C_2_-C_3_ | C_3_-O_2_ | O_2_-C_4_ | C_4_-C_5_ | C_1_-C_2_ | C_1_-S | S=O | C_2_≡N |
| --- | --- | --- | --- | --- | --- | --- | --- | --- | --- | --- |
| G1 | -1.34 | -1.31 | 0.76 |  |  |  |  |  |  |  |
| G2 | -1.32 | -1.32 | 0.68 | -1.40 |  |  |  |  |  |  |
| G3 | -1.27 | -1.15 | 0.94 | -1.16 | -1.28 | 0.88 |  |  |  |  |
| TMS |  |  | 0.13 |  |  |  | -0.49 | -2.41 | -1.34 |  |
| DMS |  |  |  |  |  |  |  | -2.10 | -1.12 |  |
| ACN |  |  |  |  |  |  | -0.32 |  |  | 6.38 |

**Supplementary Figure 2**. Reaction profile for the exergonic bond dissociations of the TMS_5_Mg^+^ complex. Free energies in eV at the PCM/wB97X-D/6-31g(d) level of theory. Each of the bond dissociation events take place after the initial Mg^+^ -> TMS charge transfer and formation of the Mg^2+^:(TMS)_5_TMS^-^ complex. While C_1_-C_2_ is slightly exergonic to break, the dissociation is precluded by the high barrier shown. The S=O and S-C bonds exhibit very similar barriers to dissociation. However, S=O dissociation is much less exergonic, so in the event of its dissociation, the bond can readily reform and lead to thermodynamically stable S-C cleavage.

# Additional computed reduction potentials

**Supplementary Table 2**. Computed reduction potentials (V vs Mg) from the PCM/wB97X-D/6-311+G(d,p) level of theory for individual solvent molecules and the following Mg^2+^ species: “bare” Mg^2+^, Mg^2+^ coordinated to one explicit solvent molecule, and pentacoordinate Mg^2+^, with alpha (the scaling factor for the atomic vDW radii used to construct the solute cavity) set to 1.1 (the Gaussian 16 default) or 1.5.

|  |  |  | **Implicit solvent and vDW radius scaling factor (alpha)** | | | | | | | | | | | |
| --- | --- | --- | --- | --- | --- | --- | --- | --- | --- | --- | --- | --- | --- | --- |
|  |  |  | Alpha=1.1 | | | | | | Alpha=1.5 | | | | | |
|  |  |  | G1 | G2 | G3 | DMS | TMS | ACN | G1 | G2 | G3 | DMS | TMS | ACN |
| **Explicitly modelled species** | Mg^2+^ | Mg^2+^ | 2.04 | 2.06 |  | 0.65 |  | 0.73 | 4.49 | 4.5 |  | 3.4 |  | 3.46 |
|  | Mg^2+^ with one explicit solvent | G1Mg^2+^ | 1.28 |  |  |  |  |  | 2.98 |  |  |  |  |  |
|  |  | G2Mg^2+^ |  | 0.87 |  |  |  |  |  | 2.22 |  |  |  |  |
|  |  | G3Mg^2+^ |  |  | 0.32 |  |  |  |  |  | 1.41 |  |  |  |
|  |  | DMSMg^2+^ |  |  |  | 0.61 |  |  |  |  |  | 2.33 |  |  |
|  |  | TMSMg^2+^ |  |  |  |  | 0.05 |  |  |  |  |  | 2.3 |  |
|  |  | ACNMg^2+^ |  |  |  |  |  | 0.36 |  |  |  |  |  | 2.57 |
|  | pentacoordinate Mg^2+^ | G1_3_Mg^2+^ | -0.07 |  |  |  |  |  | 0.68 |  |  |  |  |  |
|  |  | G2_2_Mg^2+^ |  | 0.01 |  |  |  |  |  | 0.68 |  |  |  |  |
|  |  | DMS_5_Mg^2+^ |  |  |  | -1.09 |  |  |  |  |  | -0.55 |  |  |
|  |  | ACN_5_Mg^2+^ |  |  |  |  |  | -0.87 |  |  |  |  |  | -0.34 |
|  | one solvent  molecule | G1 | -2.49 |  |  |  |  |  | -2.47 |  |  |  |  |  |
|  |  | G2 |  | -2.27 |  |  |  |  |  | -2.48 |  |  |  |  |
|  |  | G3 |  |  | -2.48 |  |  |  |  |  | -2.47 |  |  |  |
|  |  | DMS |  |  |  | -1.73 |  |  |  |  |  | -1.48 |  |  |
|  |  | TMS |  |  |  |  | -1.71 |  |  |  |  |  | -1.41 |  |
|  |  | ACN |  |  |  |  |  | -1.7 |  |  |  |  |  | -1.34 |

For individual solvent molecules, the reduction potentials are well below the Mg metal potential and do not vary significantly between the two alpha values. In contrast, the reduction potential for Mg^2+^ species is very dependent on the explicit solvation environment as well as the specified alpha. Most values of the Mg^2+^ species are positive of Mg metal, and generally increase with alpha. This is likely due to differences in the difference of electrostatic interaction between the Mg^+^ and Mg^2+^ cations and the charges on the solute cavity surface with varying distance. The values for the pentacoordinate Mg^2+^ complexes exhibit less discrepancy between the two alphas than the species with less explicit solvation, likely due to better “shielding” of the cation from the dielectric continuum by the explicit solvent molecules.

To summarize, computed reduction potentials for individual solvent molecules are relatively stable against the choice of the alpha values considered here. These solvents can confidently be considered stable against reduction by Mg metal. However, the reduction potential for Mg^2+^ species varies significantly with alpha and the degree of explicit solvation, complicating a quantitative determination of the reduction potential for solvated Mg^2+^. However, the discrepancy between alpha values decreases with increasing degree of explicit solvation for Mg^2+^ species. Upon reduction of any of the Mg^2+^ species, the cation receives the electron, and Mg^+^ species are formed. This was verified from computed NPA charges of Mg before and after reduction of all species.

# Intrinsic Reaction Coordinate (IRC) of G2_2_Mg^+^ decomposition

**Supplementary Figure 3**. IRC from TS2_G2_ to O_1_-C_2_ dissociation. The pathway involves partial dissociation of both O_1_-C_2_ and C_3_-O_2_, forming a transient radical ethylene cation before reforming the C_3_-O_2_ bond via a potential energy surface bifurcation.

**Supplementary Figure 4**. The O_1_-C_2_ and C_3_-O_2_ bond lengths during the course of the IRC in Fig S3.

# Effect of alpha on the potential energy surface and location of difficult transition states

As discussed in the text, it was noted during exploration of transition states that raising the value of alpha within the PCM method from the default allowed easier convergence of some transition state optimizations. A value of 1.5 was found to work reasonably well and thus applied to all calculations involving Mg^+^-mediated decomposition. To probe the origin of the difficulties with lower alpha values, IRC calculations were carried out on TS1_TMS_, and single point energies were obtained on points of the IRC calculations at the PCM/wB97X-D/6-311+G(d,p) level of theory with different values for alpha. The results are given in Fig S5.

**Supplementary Figure 5**. Relative energies (eV) of single point energies at the PCM/wB97X-D/6-311+G(d,p) level of theory, with different values for alpha within the PCM method, performed on steps of the IRC calculations of TS1_TMS_. Energies were set to zero at the first point. The positive and negative directions of the x-axis go towards dissociation and re-association of the C-S bond, respectively.

In Fig S5, smooth parabolic curves appear for Alpha=1.5 and 1.7, albeit with maxima slightly displaced from the origin; nonetheless, these curves appear physically reasonable and suggest that the transition state should exist on these potential energy surfaces. The same may be said for the Alpha=1.3 curve except the curve is less smooth and shows a sharp bend at the maximum. In contrast with the other curves, the curve for Alpha=1.1 shows stark irregularities and oscillations that would be indicative of a discontinuous potential energy surface. These data show that, to obtain a physically reasonable potential energy surface at this level of theory for this reaction, some value of alpha is needed higher than the default.

# Optimized cartesian coordinates

PCM/wB97X-D/6-31G(d) optimized cartesian coordinates (Å) and absolute free energies (1 M concentration) at the PCM/wB97X-D/6-311+G(d,p)//PCM/wB97X-D/6-31G(d) level of theory of the structures in Mg^+^-mediated decomposition pathways.

31

ACN_5_Mg^2+^ G=-863.565946314

Mg 0.0028760 0.0013110 -0.8143460

H -3.4622860 3.7911710 -1.7070220

C -2.2242000 2.5279400 -0.5852170

C -3.1882180 3.6153690 -0.6633760

N -1.4592300 1.6647860 -0.5478570

H -2.7505290 4.5263220 -0.2481530

H -4.0845150 3.3568480 -0.0945430

H -0.1528980 1.0155430 4.4276030

C 0.0042290 -0.0021370 2.6038380

C 0.0097740 -0.0002510 4.0597540

N -0.0003840 -0.0035370 1.4516900

H 0.9726590 -0.3652620 4.4248980

H -0.7859360 -0.6496720 4.4322600

H 2.8529040 -4.4511940 -0.0324940

C 2.2219600 -2.5328750 -0.5908240

C 3.1802970 -3.6253330 -0.6685230

N 1.4625810 -1.6648320 -0.5528600

H 4.1605220 -3.2798460 -0.3313800

H 3.2579430 -3.9752020 -1.7013530

H -3.9461040 -3.2935720 -1.6989800

C -2.5335250 -2.2188160 -0.5854210

C -3.6278560 -3.1756090 -0.6599250

N -1.6635560 -1.4612550 -0.5518310

H -4.4714060 -2.8196440 -0.0634960

H -3.2977610 -4.1426830 -0.2728410

H 4.5198910 2.7654020 -0.2192290

C 2.5304940 2.2246270 -0.5866000

C 3.6179000 3.1883170 -0.6679540

N 1.6672260 1.4600190 -0.5464130

H 3.3444750 4.1001760 -0.1317800

H 3.8169350 3.4322120 -1.7149540

31

TS1_ACN_ G=-863.5604213

Mg -0.0000350 0.1909880 -0.4593510

H 5.0936230 0.5086840 -1.4853790

C 3.3585290 0.0777710 -0.3940470

C 4.8085120 0.0947290 -0.5143920

N 2.2066560 0.0727670 -0.3231170

H 5.1976890 -0.9226400 -0.4289010

H 5.2381420 0.7107360 0.2792200

H 0.8894090 -0.4238540 4.7229890

C -0.0017570 -0.5383070 2.8324970

C -0.0023710 -0.8470660 4.2547220

N -0.0012570 -0.2917390 1.7072030

H -0.0043800 -1.9304480 4.3959020

H -0.8926030 -0.4205570 4.7229400

H -5.2416470 0.6574390 0.3251800

C -3.3585360 0.0885150 -0.3952100

C -4.8084590 0.1101470 -0.5154790

N -2.2066890 0.0797230 -0.3242700

H -5.1934580 -0.9123950 -0.5141110

H -5.0940780 0.6008970 -1.4498240

H 0.0086710 4.5148760 -2.1701380

C 0.0064040 3.4298570 -0.3685810

C 0.0090370 4.7029470 -1.0861060

N 0.0040210 2.2727440 -0.1867060

H 0.8963230 5.2839890 -0.8231420

H -0.8758630 5.2876320 -0.8231740

H -0.8962680 -4.9430760 -1.4091570

C -0.0045590 -3.0539840 -1.2727090

C -0.0065490 -4.4212020 -1.7695590

N -0.0029820 -1.9625810 -0.8987950

H 0.8848780 -4.9437450 -1.4143890

H -0.0097630 -4.4162310 -2.8628190

37

ACN_5_ACN^-^Mg^2+^ G=-996.306373015

Mg -0.0116870 0.0001890 0.0682800

H -4.2972640 0.9185500 -2.5481460

C -2.5323170 0.0159390 -1.8550990

C -3.9783020 0.0231390 -2.0092200

N -1.3859240 0.0101820 -1.7118070

H -4.3965560 0.0225990 -0.9982000

H -4.3056950 -0.8665050 -2.5526270

H -4.4462400 -0.8632490 2.3890630

C -2.7924630 0.0109410 1.3136480

C -3.7929160 0.0137450 2.4613240

N -1.5682030 0.0057590 1.3634900

H -4.4389860 0.8962640 2.3911230

H -3.2982950 0.0105480 3.4443830

H 3.9853820 -0.9769040 3.4101590

C 2.4212500 -0.0191510 2.4003770

C 3.4430590 -0.0288320 3.4369680

N 1.6101930 -0.0116540 1.5825720

H 2.9740710 0.0904650 4.4164960

H 4.1445080 0.7920780 3.2705600

H 1.0213670 -5.2079420 0.3205300

C 0.1339620 -3.3618180 -0.1146680

C 0.1275920 -4.8163480 -0.1708680

N 0.1375420 -2.2109160 -0.0691180

H 0.1165370 -5.1445620 -1.2128890

H -0.7602450 -5.1998460 0.3373690

H 4.0365460 0.9973440 -3.2317490

C 2.5324280 -0.0061820 -2.1760540

C 3.6154960 -0.0073730 -3.1486550

N 1.6755380 -0.0050640 -1.4064940

H 3.2341750 -0.3198990 -4.1236000

H 4.3972540 -0.7001610 -2.8284610

H -0.2737280 5.1559480 -1.0832430

C 0.1843110 4.8159910 -0.1514160

H 1.2139660 5.1780620 -0.1032490

H -0.3791470 5.2174300 0.6941610

C 0.1718780 3.3612590 -0.1017550

N 0.1606720 2.2102150 -0.0612910

37

TS2_ACN_ G=-996.2790159

Mg -0.1897440 0.0698000 -0.0660560

H 2.5193610 4.1456330 0.9179670

C 1.6789730 2.2362240 1.1572950

C 2.7811550 3.0962640 0.7653100

N 0.8000280 1.5235960 1.3937270

H 2.9634970 2.9054890 -0.2996480

H 3.6771250 2.8530580 1.3408040

H 4.7557670 0.7945500 -1.6455170

C 2.1163890 1.1099580 -2.0029810

C 3.9406280 0.1997840 -1.2455870

N 1.1050140 0.7519170 -1.4993210

H 3.8255840 -0.7912740 -1.6765150

H 3.7854270 0.2699870 -0.1708630

H -2.2832220 -4.1229540 -2.4972480

C -1.7304870 -2.1584410 -2.0336280

C -2.3623280 -3.1203160 -2.9239820

N -1.2293420 -1.3967760 -1.3298180

H -1.8650960 -3.1012530 -3.8966330

H -3.4167840 -2.8647030 -3.0521900

H 3.3992240 -3.7877570 0.4402290

C 2.2469760 -2.0598210 0.7182010

C 3.4882630 -2.7972210 0.8922960

N 1.2805420 -1.4519180 0.5630870

H 3.7135320 -2.9031750 1.9559650

H 4.2945760 -2.2437350 0.4011410

H -3.9792510 -1.4216210 3.3066870

C -2.1065490 -0.9887550 2.4784010

C -2.9253710 -1.4376560 3.5943020

N -1.4582280 -0.6323070 1.5959670

H -2.7743650 -0.7750820 4.4497880

H -2.6422150 -2.4557420 3.8716510

H -3.5626590 4.1178580 -0.1381140

C -3.5209670 3.4071220 -0.9668880

H -4.5043520 2.9502240 -1.1003010

H -3.2405460 3.9348500 -1.8814810

C -2.5367620 2.3755010 -0.6767000

N -1.7563300 1.5600860 -0.4478250

37

ACN_6_Mg^2+^ C-C dissociated G=-996.29676159

Mg -0.1671090 0.0617340 -0.1404500

H 2.3843340 4.2536140 0.6063450

C 1.7778320 2.2571210 0.8531040

C 2.6905060 3.2473910 0.3117260

N 1.0369190 1.4424630 1.2055220

H 2.6472020 3.1432700 -0.7800710

H 3.7083890 3.0490180 0.6553470

H 5.3704520 0.3248160 -0.4956620

C 1.5017170 1.4966900 -2.4869280

C 4.3297660 0.2778040 -0.1975550

N 0.8260460 0.8856330 -1.7431890

H 3.5408570 0.4156180 -0.9296840

H 4.0792880 0.1597900 0.8511290

H -2.8232920 -3.9110610 -2.3999900

C -2.0699080 -2.0083800 -1.9610350

C -2.8759890 -2.8918030 -2.7898190

N -1.4314430 -1.3084380 -1.3059920

H -2.4995840 -2.8770770 -3.8153670

H -3.9154580 -2.5554580 -2.7817840

H 3.2254540 -3.9445370 -0.5597640

C 2.2250050 -2.2446310 0.1442120

C 3.3675500 -3.1439700 0.1699190

N 1.3324230 -1.5171850 0.1177310

H 3.4777680 -3.5767580 1.1669240

H 4.2666350 -2.5744430 -0.0823780

H -3.3344260 -1.6212640 3.7482400

C -1.6303570 -1.1705370 2.6197250

C -2.2474530 -1.6976590 3.8273860

N -1.1408710 -0.7528070 1.6647300

H -1.9072080 -1.1246250 4.6931460

H -1.9674520 -2.7459230 3.9554060

H -4.3205730 3.2805810 0.5427190

C -3.5939180 3.4263170 -0.2599260

H -4.1065420 3.3835950 -1.2238160

H -3.1212570 4.4047550 -0.1461060

C -2.5798830 2.3846060 -0.1995180

N -1.7757170 1.5614210 -0.1540860

56

DMS_5_Mg^+^ G=-3341.746951

Mg 0.0340340 -0.1672220 -1.3095980

S 1.8790530 2.4054430 0.1955690

O 1.1984530 2.3483590 1.4902880

C 1.5774420 3.9913840 -0.5464300

H 2.1104330 4.0459380 -1.4968300

H 0.4963520 4.0574670 -0.7057660

H 1.9124230 4.7691110 0.1423490

H 4.1468390 2.5270200 -0.4879060

O 1.5527790 1.3767710 -0.8248360

C 3.6366060 2.3187420 0.4542890

H 3.9171960 3.0436580 1.2199120

H 3.8515910 1.2963520 0.7758670

O -1.7156240 3.7302440 -0.7311750

H -4.4845050 3.0950730 -0.3601440

H -2.7377570 2.8572790 1.7932410

C -3.9497510 2.2931500 -0.8715980

S -2.2220650 2.3859730 -0.4587670

H -4.0259220 2.4156620 -1.9539030

C -2.1638990 2.0787830 1.2872720

H -1.1088010 2.1230180 1.5705340

H -4.3191240 1.3126130 -0.5640360

H -2.5761790 1.0844290 1.4609610

O -1.5768750 1.2594010 -1.1688120

H 2.3681190 -0.9671640 2.0433490

C 1.5805860 -0.7119030 2.7576010

O -0.0091600 -2.5961900 1.7641560

O -0.1864700 -0.2441830 0.8618910

S 0.0349040 -1.1554720 2.0043820

H 1.6965400 -1.2809730 3.6816150

H 1.5749320 0.3648290 2.9382210

C -1.1975640 -0.7446040 3.2209770

H -2.1639880 -0.9575390 2.7576430

H -1.1162270 0.3121750 3.4802860

H -1.0457280 -1.3743370 4.0990690

H -1.8814690 -3.5660410 0.8318960

C -2.7049030 -3.5806180 0.1115040

O -3.1939440 -1.0060280 0.4806250

O -1.4878510 -1.7109090 -1.2643610

H -3.6547620 -3.8039560 0.5999730

S -2.7728360 -1.9400580 -0.5682090

H -2.4928560 -4.2890630 -0.6908200

C -4.0329040 -1.9824210 -1.8241410

H -4.9948740 -2.2051720 -1.3595980

H -4.0457400 -0.9894860 -2.2788020

H -3.7691480 -2.7310220 -2.5726210

H 4.7608840 -0.7769450 -2.3305040

C 3.6692100 -0.7675320 -2.3404550

H 3.2820670 0.2521660 -2.2880550

H 4.7283040 -3.2918420 -1.0435500

H 3.2688880 -1.2691010 -3.2229990

C 3.6372670 -3.2773410 -1.0306470

O 3.7075420 -0.9871430 0.2944540

S 3.0791770 -1.5965500 -0.8815430

H 3.2217370 -3.7113980 -1.9413780

H 3.2588540 -3.8064130 -0.1534180

O 1.6023080 -1.6484350 -0.9275490

56

TS1_DMS_ G=-3341.733361

Mg 0.0122190 -0.0249280 -1.1899220

S 1.5380050 2.6001710 -0.0269300

O 1.0363390 2.7216410 1.3836810

C 1.0058840 4.0927080 -0.8565640

H 1.4409130 4.1146490 -1.8573270

H -0.0848050 4.0608740 -0.9211220

H 1.3234640 4.9538950 -0.2633540

H 3.6776420 2.9417740 -1.0074840

O 1.3035730 1.4926060 -1.1453790

C 3.3191350 2.7855390 0.0122900

H 3.5728760 3.6328550 0.6534430

H 3.7297040 1.8561160 0.4116110

O -2.3991770 3.5517670 -0.6935640

H -4.9058340 2.3196020 -0.0066640

H -2.9296960 2.5898150 1.9478920

C -4.2552940 1.6495410 -0.5712200

S -2.5620530 2.1441520 -0.3405070

H -4.4650460 1.7294970 -1.6398550

C -2.2625190 1.9259280 1.3950660

H -1.2133830 2.1909980 1.5657230

H -4.3562390 0.6159480 -0.2329360

H -2.4447910 0.8768600 1.6299370

O -1.7501810 1.1555720 -1.0863000

H 2.5232920 -0.2229670 2.2488280

C 1.6470270 -0.0854250 2.8862000

O 0.7588760 -2.3834030 1.8884540

O -0.1258940 -0.2256280 0.9330410

S 0.3408940 -0.9984030 2.1072540

H 1.8177290 -0.5030750 3.8802200

H 1.3654790 0.9694710 2.9123080

C -1.0067240 -0.9802000 3.2695900

H -1.8687210 -1.4043830 2.7481960

H -1.2094830 0.0493800 3.5702010

H -0.7340390 -1.5893660 4.1326460

H -0.7679720 -3.4661670 0.6270460

C -1.6258270 -3.7695240 0.0180300

O -2.9165320 -1.4980320 0.4838400

O -1.3301830 -1.6361690 -1.4889640

H -2.3687720 -4.2918690 0.6233520

S -2.3475940 -2.2760030 -0.6194990

H -1.2931770 -4.3838450 -0.8203080

C -3.6608780 -2.7979050 -1.6986990

H -4.4064660 -3.3398050 -1.1145570

H -4.0947130 -1.8898350 -2.1224770

H -3.2467640 -3.4218060 -2.4920070

H 4.8027050 -0.6635040 -2.1587870

C 3.7235550 -0.7312440 -2.3084510

H 3.2747990 0.2581740 -2.4358160

H 4.6746550 -3.0178560 -0.5196880

H 3.4739160 -1.3689710 -3.1579900

C 3.5927080 -3.0510680 -0.6573480

O 3.4165820 -0.5979290 0.3088970

S 2.9769550 -1.3941390 -0.8377190

H 3.3194990 -3.6353480 -1.5372130

H 3.1027800 -3.4511410 0.2335630

O 1.5243590 -1.5370990 -1.0928610

67

DMS_5_DMS^-^Mg^2+^ G=-3970.144643

O -1.1902430 2.6797530 2.8237050

S -1.8160380 2.0615540 1.6559580

O -0.9290380 1.7384150 0.5152390

O -2.2269900 -3.1374330 -1.2998480

O -1.1814990 -1.3099330 0.0612510

S -1.7171340 -2.6867800 -0.0048130

O -3.3122070 0.6485000 -1.2055370

O -0.9331800 0.5630640 -2.0888600

S -2.3880160 0.6344680 -2.3411620

O 2.4711330 -3.1580430 -0.6266370

S 2.5924460 -1.9096980 -1.3826770

O 1.3477120 -1.1413070 -1.6302650

C -2.6898740 0.5939020 2.1317500

C -3.0721200 3.1399690 1.0064960

C -2.6469200 2.1073030 -3.3069560

C -2.8041040 -0.7181960 -3.4140810

C 3.2569310 -2.2712940 -2.9925550

C 3.7779560 -0.8520740 -0.5970270

C -0.4833170 -3.8133020 0.5882570

C -2.9955770 -2.7772150 1.2250830

Mg 0.2814580 0.1911980 -0.3559000

S 0.6819260 -0.9919830 2.5245390

O -0.4722360 -1.8084150 3.2113680

C 1.0643600 0.2194520 3.8072900

O 1.4405900 -0.1071750 1.2316660

C 2.0687430 -2.1355560 2.7040360

H 0.3746340 1.0595460 3.6959530

H 0.9200600 -0.2612550 4.7763150

H 2.0942270 -2.7764770 1.8201720

H 1.9104480 -2.7260640 3.6077860

O 1.4681790 1.7934500 -1.0966050

O 3.8637920 2.4199690 -0.6865060

S 2.4489840 2.7295580 -0.4968510

C 2.1001640 2.8862050 1.2356110

C 2.0933140 4.3220140 -1.2068870

H 2.2930600 4.2408500 -2.2772310

H 2.7488100 5.0681070 -0.7546110

H 1.0431880 4.5614720 -1.0339670

H 2.8651020 3.5193720 1.6875400

H 2.1215710 1.8573720 1.6151420

H 1.0981970 3.2947710 1.3743850

H 3.9612950 0.0236590 -1.2220640

H 4.6902930 -1.4252860 -0.4226830

H 3.2974310 -0.5429870 0.3400180

H 3.3871890 -1.3378240 -3.5420250

H 2.5288430 -2.9102860 -3.4962260

H 4.2057860 -2.7970130 -2.8746240

H -2.5658390 4.0448770 0.6637460

H -3.7787920 3.3827130 1.8017550

H -3.5605870 2.6273020 0.1751630

H -3.1614480 0.1974030 1.2314940

H -3.4259090 0.8644040 2.8914000

H -1.9616790 -0.1198560 2.5367770

H 0.4121270 -3.6887890 -0.0269850

H -0.8793400 -4.8266920 0.5002140

H -0.2859220 -3.5444590 1.6299250

H -3.7911850 -2.0975320 0.9137970

H -2.5465610 -2.4744040 2.1758010

H -3.3722400 -3.8008620 1.2645820

H -2.1486890 -0.6912130 -4.2860000

H -3.8530490 -0.6281590 -3.7017720

H -2.6383220 -1.6315290 -2.8335810

H -2.3642180 2.9477590 -2.6697400

H -3.7004090 2.1747960 -3.5827420

H -2.0065470 2.0751850 -4.1896000

H 2.0913320 0.5673560 3.6876160

H 2.9995310 -1.5685400 2.7572690

67

TS2_DMS_ G=-3970.136777

O -2.0258280 2.3992080 2.4886160

S -2.3927910 1.4100250 1.4774140

O -1.4770310 1.2856440 0.3135730

O -0.6267740 -4.0691900 -0.4407750

O -0.5996620 -1.6956960 0.3435360

S -0.3559020 -3.1268830 0.6450620

O -3.3543810 -0.8958730 -1.0350240

O -1.1459070 -0.2269980 -2.0840030

S -2.4811380 -0.8540240 -2.2087600

O 3.6722190 -1.9711770 -0.5610750

S 3.1093400 -0.9619850 -1.4564330

O 1.6308030 -0.9069550 -1.5742700

C -2.6042280 -0.1875090 2.2132710

C -3.9853470 1.8092730 0.7947800

C -3.3108470 0.0292190 -3.5118940

C -2.2477070 -2.4996070 -2.8350060

C 3.7048470 -1.2423360 -3.1090030

C 3.7205920 0.6404690 -0.9972470

C 1.3000780 -3.3351700 1.2380480

C -1.3707220 -3.5066450 2.0524510

Mg 0.1619000 0.1106460 -0.4264700

S 1.0528910 0.1647170 2.6555680

O 0.4256360 -1.0905230 3.2924090

C 1.0252130 1.6202060 3.8818520

O 1.4487920 0.4461370 1.0575680

C 2.7936770 -0.2322840 3.0150570

H -0.0085290 1.9632760 3.9369860

H 1.3904720 1.2782880 4.8539310

H 3.0859740 -1.0542240 2.3588360

H 2.8802790 -0.5305300 4.0618670

O 0.7631920 1.8650700 -1.4406740

O 2.0401320 3.9825800 -1.7649060

S 0.9119700 3.3070970 -1.1352520

C 1.0001310 3.5261390 0.6274740

C -0.6167970 4.0787740 -1.6152520

H -0.6908110 3.9811110 -2.7002220

H -0.5986020 5.1315010 -1.3284310

H -1.4321910 3.5426360 -1.1272810

H 1.1508070 4.5881540 0.8285470

H 1.8496780 2.9309900 0.9651330

H 0.0874300 3.1497740 1.0925250

H 3.3867880 1.3814820 -1.7254190

H 4.8091870 0.5874350 -0.9409560

H 3.2700520 0.8353090 -0.0177890

H 3.3168710 -0.4611760 -3.7645000

H 3.3258290 -2.2189400 -3.4167810

H 4.7959620 -1.2451200 -3.0982670

H -3.8898990 2.7831890 0.3099860

H -4.7141330 1.8658520 1.6050330

H -4.2467600 1.0353940 0.0700240

H -2.9094130 -0.8728840 1.4205970

H -3.3617510 -0.1028850 2.9950680

H -1.6346910 -0.4805150 2.6360730

H 1.9880790 -2.9721660 0.4703340

H 1.4547310 -4.3999400 1.4232520

H 1.3847720 -2.7441380 2.1538200

H -2.4094470 -3.3639970 1.7476660

H -1.0860550 -2.8194240 2.8533960

H -1.2022070 -4.5473740 2.3347630

H -1.6487980 -2.4446630 -3.7455840

H -3.2268780 -2.9403540 -3.0306950

H -1.7170970 -3.0655040 -2.0626470

H -3.4490700 1.0520770 -3.1558930

H -4.2777070 -0.4397780 -3.7011590

H -2.6850740 0.0232450 -4.4054590

H 1.6702170 2.4084040 3.4850440

H 3.4264200 0.6335750 2.8072510

67

DMS_6_Mg^2+^ C-S dissociated G=-3970.17989805

O 2.9864290 1.8105930 -1.8586150

S 2.8487310 1.0142710 -0.6423820

O 1.5555070 1.1483620 0.0802690

O -0.0519690 -3.9775990 1.3270220

O 0.4338210 -1.7486550 0.3166260

S 0.1552950 -3.1917380 0.1111630

O 2.5352290 -0.7080980 2.4585020

O 0.1742760 0.2121700 2.3911770

S 1.2750990 -0.4292630 3.1472840

O -3.9215460 -1.5996120 -0.6007940

S -3.5880300 -0.4713950 0.2657500

O -2.2533950 -0.4883790 0.9156750

C 3.1116300 -0.7025820 -0.9750240

C 4.1095270 1.4659240 0.5261130

C 1.5968180 0.6458480 4.5281060

C 0.6410000 -1.9275410 3.8612480

C -4.7621280 -0.3769170 1.5979630

C -3.7817370 1.0438730 -0.6409040

C -1.2152710 -3.3790440 -0.9923460

C 1.5341640 -3.8361190 -0.8051740

Mg -0.3437410 0.1996360 0.3229100

S -0.2149860 -0.1592350 -2.9488560

O 0.5152090 -1.4799630 -2.7811030

C 3.4541090 -0.6022340 -4.4016400

O -0.8792490 0.2716570 -1.5992860

C -1.7223300 -0.6365160 -3.8349120

H 3.5962620 0.4010430 -4.0166550

H 2.5487300 -1.1445320 -4.1511380

H -2.2919190 -1.3146870 -3.1924660

H -1.4433020 -1.1292140 -4.7688920

O -1.1043930 2.1413120 0.6241740

O -2.0844520 4.3753620 0.1166700

S -0.9117740 3.5098090 0.0886710

C -0.2933130 3.4063300 -1.5754890

C 0.4128220 4.2335660 1.0284920

H 0.0500910 4.3326470 2.0535990

H 0.6580270 5.2133890 0.6154610

H 1.2648920 3.5535090 0.9889040

H -0.1733910 4.4236510 -1.9521820

H -1.0443910 2.8646650 -2.1515560

H 0.6490870 2.8568800 -1.5906860

H -3.6442950 1.8930150 0.0299530

H -4.7735320 1.0419520 -1.0961740

H -2.9925920 1.0261910 -1.3973740

H -4.5299420 0.4915570 2.2162030

H -4.6518720 -1.2957850 2.1773700

H -5.7683070 -0.3100820 1.1810010

H 3.9675070 2.5240880 0.7558910

H 5.0869650 1.3125560 0.0655320

H 3.9815540 0.8507380 1.4190740

H 3.0726800 -1.2295550 -0.0197980

H 4.0736950 -0.8171980 -1.4780420

H 2.2916050 -1.0164390 -1.6343800

H -2.0909760 -2.9124240 -0.5371910

H -1.3802500 -4.4477790 -1.1426430

H -0.9351900 -2.8685570 -1.9178120

H 2.4246140 -3.6992260 -0.1885240

H 1.6005720 -3.2738120 -1.7395220

H 1.3647800 -4.8986980 -0.9877950

H -0.2683930 -1.6915310 4.4164080

H 1.4050960 -2.3516050 4.5152110

H 0.4182880 -2.6119940 3.0370960

H 1.9658000 1.5852800 4.1115330

H 2.3563210 0.1902440 5.1653730

H 0.6688200 0.8156600 5.0759470

H 4.1966180 -1.0418380 -5.0571890

H -2.3135940 0.2583390 -4.0466680

76

TMS_5_Mg^+^ G=-3728.65508

Mg 0.0022750 0.5360340 -1.1713480

S -2.2636390 -1.8953600 -0.0104420

O -1.3917630 -2.4036770 1.0505980

C -2.8038530 -3.2039500 -1.1478450

O -1.8025180 -0.7394060 -0.8260510

C -3.9000500 -1.4871570 0.6342350

O 0.1627470 -3.4463060 -1.7108080

S 1.3090730 -2.5991440 -1.3815170

O 1.2086090 -1.1524270 -1.7037910

O 1.3668480 1.7678820 2.3223350

O 0.3419170 -0.0315670 0.8770040

S 0.5261130 0.5759310 2.2125540

O 3.3967930 0.0487690 0.3992310

O 1.8539510 1.6121410 -0.8885640

S 3.1934790 1.3033940 -0.3294370

O -3.4516850 1.7764090 0.8544410

S -2.6734010 2.3549990 -0.2470820

O -1.2066460 2.1283480 -0.2309380

C -4.3029630 -3.3762870 -0.8794650

H -4.8080380 -3.7453630 -1.7745610

H -4.4577990 -4.1114840 -0.0831220

H -2.1918210 -4.0826680 -0.9478300

H -2.5856560 -2.8356610 -2.1515490

C -4.8569280 -2.0112230 -0.4353300

H -4.8889750 -1.3207730 -1.2853820

H -5.8698690 -2.0953250 -0.0364060

H -3.9360160 -0.4101190 0.8138380

H -4.0058130 -2.0342100 1.5751920

H -4.9718620 3.3189040 -2.1195200

H -3.8713570 4.3524000 0.1437170

H -3.5386020 5.3266450 -2.0999290

C -3.9313480 3.1962150 -2.4371480

C -2.9442000 4.1322840 -0.3920010

C -3.0779170 4.3575740 -1.8978450

H -3.9172130 3.1620110 -3.5287250

C -3.3507150 1.8992390 -1.8637000

H -4.0747770 1.0991190 -1.7080710

H -2.1037640 4.6402240 0.0831730

H -2.0876860 4.3487850 -2.3656660

H -2.4972030 1.5206690 -2.4444820

H 2.8616240 -4.7123820 0.1951120

H 2.6123580 -4.2294500 -2.4862950

H 4.6958980 -3.8514420 -1.2408840

C 3.1068520 -3.6468730 0.2592610

C 2.8297770 -3.2146180 -2.1431660

C 3.8544270 -3.1958220 -1.0072760

H 3.7115800 -3.4951220 1.1559330

C 1.8312980 -2.8060770 0.3363180

H 0.9935710 -3.2501810 0.8754580

H 3.0648940 -2.5739010 -2.9940380

H 4.2369100 -2.1803530 -0.8638390

H 2.0337750 -1.7952420 0.6934610

H 5.8894890 2.7183290 0.2968190

H 5.2028520 0.7685420 -1.4577870

H 5.7772610 3.0644620 -2.1408620

C 5.0090610 3.2379800 -0.0952340

C 4.3886750 1.4649440 -1.6754860

C 4.8437580 2.9216670 -1.5926500

H 5.1506330 4.3069780 0.0771100

C 3.7476540 2.7455540 0.6221810

H 3.8936700 2.4201890 1.6519050

H 3.8877310 1.1841220 -2.6030770

H 4.0872980 3.5754140 -2.0391620

H 2.9241440 3.4604980 0.5830110

H -1.6220080 -1.2031110 3.4234660

H 0.9375300 -1.6749820 2.7880850

H 0.1651680 -1.5724480 5.0989590

C -1.2434730 -0.3309850 3.9665250

C 1.0859130 -0.7365320 3.3254900

C 0.1531540 -0.6387390 4.5328180

H -1.9566000 -0.0807890 4.7549080

C -1.0802050 0.8465350 3.0030650

H -1.8436460 0.9225550 2.2280400

H 2.1451430 -0.5743450 3.5302880

H 0.4787090 0.1646730 5.2021650

H -0.9943480 1.8016160 3.5273690

76

TS1_TMS_ G=-3728.641811

Mg 0.0530590 0.2264580 -1.0458640

S -2.6685230 -1.4026650 -0.1699830

O -2.4950670 -1.8891420 1.2372830

C -2.7462060 -2.8388030 -1.2980790

O -1.8614730 -0.3222540 -1.0223260

C -4.3977930 -0.9107770 -0.4202680

O 0.2514150 -3.5668060 -0.2295680

S 1.2818910 -2.8728290 -1.0013500

O 0.8971620 -1.6140600 -1.6975430

O 1.7892670 -0.0337210 3.0519370

O 0.6567750 -0.4510550 0.8687170

S 0.5963500 -0.5164490 2.3532410

O 4.0005320 0.3910110 -0.2417250

O 1.8820180 1.1979360 -1.3515360

S 3.0858330 1.4962980 -0.5253850

O -2.8889980 1.9849620 0.9688150

S -1.9762950 2.5993330 0.0038970

O -0.5766630 2.0838770 -0.0496550

C -4.2051890 -2.9443400 -1.7598160

H -4.2622580 -3.4088750 -2.7472900

H -4.7726830 -3.5714710 -1.0639030

H -2.3743480 -3.7014780 -0.7454300

H -2.0627750 -2.6078470 -2.1155690

C -4.7881660 -1.5222610 -1.7626180

H -4.3558160 -0.9349760 -2.5801320

H -5.8738590 -1.5275660 -1.8870630

H -4.4516860 0.1760100 -0.3582910

H -4.9557810 -1.3544900 0.4089230

H -3.7493820 4.5264070 -1.8000240

H -2.7568300 4.7145920 0.7097180

H -1.8716250 5.9874530 -1.2267660

C -2.7489240 4.1490450 -2.0336480

C -1.8329810 4.3813630 0.2288840

C -1.7030460 4.9091300 -1.1980350

H -2.5823160 4.2893930 -3.1038400

C -2.6537060 2.6594290 -1.6768260

H -3.6083730 2.1335170 -1.6691420

H -0.9815760 4.5759680 0.8825160

H -0.6944680 4.7148210 -1.5782550

H -1.9452550 2.0932230 -2.2978240

H 4.1074460 -4.2833270 -0.4077680

H 1.9538390 -4.9423910 -1.9283340

H 4.1168440 -4.1184190 -2.8403410

C 3.9223050 -3.2879360 -0.8227360

C 2.0359010 -3.9060500 -2.2680320

C 3.4828640 -3.4176210 -2.2932980

H 4.8433400 -2.7086950 -0.7336980

C 2.7949290 -2.5956590 -0.0402330

H 2.6268930 -3.0172840 0.9516290

H 1.4785050 -3.7664700 -3.1952530

H 3.5375680 -2.4470800 -2.7967580

H 2.9384060 -1.5165250 0.0313870

H 4.3482420 3.5764110 1.3378720

H 5.0068010 2.7088010 -1.1649080

H 4.1196650 4.9519320 -0.7067980

C 3.3438860 3.7034520 0.9207910

C 3.9378540 2.8798330 -1.3159510

C 3.4385540 4.1114100 -0.5593230

H 2.8309340 4.4607080 1.5173650

C 2.5789320 2.3777160 0.9743540

H 2.8031200 1.7372030 1.8287780

H 3.7002570 2.8581270 -2.3802550

H 2.4532450 4.4072240 -0.9350450

H 1.4978590 2.5165470 0.8907570

H -2.4113590 -1.1456640 3.4842060

H -0.4620460 -2.6076280 2.1329990

H -1.0486450 -2.8095090 4.5483910

C -1.5171090 -0.7526940 3.9694130

C 0.1943220 -2.1761540 2.8934750

C -0.5249870 -1.9111840 4.2154400

H -1.8045750 -0.2807980 4.9109370

C -0.8784590 0.2826210 3.0215580

H -1.5321520 0.5568560 2.1927010

H 1.1242410 -2.7401870 2.9832480

H 0.2054570 -1.6424750 4.9852710

H -0.5172740 1.1823190 3.5241370

91

TMS_5_TMS^-^Mg^2+^ G=-4434.437504

O -0.3121760 4.0624420 -0.8852280

S -1.2135560 2.9115410 -0.9642980

O -0.5783890 1.6067750 -1.2702810

O -2.9809100 -2.0161950 2.1044650

O -1.6418060 -0.2750180 0.8956170

S -2.0395410 -0.8998790 2.1883740

O -3.3770220 0.1200210 -1.7831610

O -1.1334470 -1.0460300 -1.9187900

S -2.6135930 -1.1055060 -2.0177040

O 0.8444030 -4.0397100 1.9158760

S 1.3109500 -3.2086070 0.8072750

O 0.3097370 -2.2796880 0.2197700

H -4.3492610 2.4229710 -1.2976540

H -2.5726760 1.7664020 0.5630540

H -2.5841480 2.4089970 -2.8479290

C -3.8235350 3.3811120 -1.2986170

C -2.2548730 2.8098510 0.4946050

C -2.5476450 3.2424130 -2.1461490

H -4.2427540 3.5969430 0.8390700

C -3.4132700 3.7396280 0.1430860

H -4.4855960 4.1363210 -1.7271110

H -1.6555000 3.0749530 1.3704530

H -2.2647030 4.1643520 -2.6584840

H -3.0938240 4.7849180 0.2113250

H -4.8823070 -3.2269930 -2.1666250

H -3.9425080 -1.4112700 -3.9474510

H -3.8717060 -2.1180290 -0.2553670

C -3.8258680 -3.4745470 -2.0198560

C -2.9824090 -1.8270310 -3.6304100

C -3.1945590 -2.5017150 -1.0177260

H -3.5822550 -3.8401990 -4.1674440

C -3.0689950 -3.3271220 -3.3515380

H -3.7745120 -4.4961180 -1.6376110

H -2.1943520 -1.5249100 -4.3215840

H -2.3056930 -2.9074800 -0.5301230

H -2.0624850 -3.7500710 -3.2675030

H 4.3386090 -3.8217750 1.0138680

H 2.1795740 -5.2041760 -0.0966290

H 2.9305670 -2.3571640 2.3099510

C 3.9312200 -2.9852700 0.4364730

C 2.0141830 -4.2105480 -0.5210060

C 2.8147940 -2.2933850 1.2259820

H 3.9967600 -4.1861060 -1.3939060

C 3.3202100 -3.5016340 -0.8776590

H 4.7347980 -2.2748310 0.2387380

H 1.2820320 -4.2630470 -1.3281540

H 2.6478130 -1.2509290 0.9288870

H 3.1117030 -2.6611880 -1.5427800

H -2.9464610 -1.0837950 4.9119510

H -0.5885430 -2.4498300 3.2119280

H -3.7265060 0.4822500 3.1292430

C -2.2909330 -0.2399970 4.6733800

C -0.6030820 -1.3588190 3.1882070

C -2.6588570 0.3498980 3.3127090

H -0.5476730 -1.3750070 5.3645410

C -0.8191180 -0.6898000 4.5592470

H -2.4079560 0.5128740 5.4553190

H 0.2784640 -0.9964490 2.6564470

H -2.0806870 1.2622240 3.1233790

H -0.1885880 0.1996830 4.6204510

Mg 0.0861740 -0.2276770 -0.3867060

S 0.8789460 1.6662870 2.1324320

O -0.1180220 2.2535090 3.1910510

C 1.7697230 3.1543810 1.5385880

O 1.3788000 0.6036870 0.8375360

C 2.2722420 1.1119310 3.1768270

C 3.5222600 1.6553020 2.4945610

C 3.1946990 3.1076280 2.1168970

H 3.9198100 3.5131410 1.4048330

H 3.2396740 3.7317850 3.0156880

H 4.3873490 1.6020710 3.1622550

H 3.7364480 1.0626130 1.6009610

H 1.7415580 3.1363120 0.4535310

H 1.1931330 4.0107590 1.8887040

H 2.2530280 0.0238970 3.2559710

H 2.1136620 1.5608870 4.1611210

O 1.6572410 -0.4606930 -1.8054290

O 4.0674270 -0.2021340 -1.0981780

S 2.9506490 0.2668320 -1.9169960

H 1.6117660 1.3969100 -4.2870370

H 1.6298250 2.1729180 -1.4868910

H 2.9771240 -0.6568870 -4.1101380

C 2.6922330 1.5481000 -4.1913770

C 2.6766100 2.0491410 -1.7637800

C 3.3528830 0.2705300 -3.6756040

H 2.3908070 3.5326150 -3.3118390

C 2.9914220 2.6359110 -3.1450710

H 3.0845720 1.8145370 -5.1749640

H 3.3240480 2.4017810 -0.9607800

H 4.4428420 0.3104560 -3.7527960

H 4.0462530 2.9226570 -3.2023030

91

TS2_TMS_ G=-4434.432049

O 0.0774770 -4.0095690 -0.3623140

S 1.0731660 -2.9432190 -0.4969540

O 0.5783930 -1.6429340 -1.0142710

O 2.6355690 2.2211760 2.3500270

O 1.4715600 0.3615410 1.1383620

S 1.7035310 1.0951100 2.4155450

O 3.5026180 -0.4300180 -1.2465630

O 1.4251970 0.9304890 -1.7419830

S 2.9053840 0.8403660 -1.6587520

O -1.2769210 4.0752760 1.6314050

S -1.4618930 3.2825910 0.4179030

O -0.3611010 2.3431900 0.0716150

H 4.2643150 -2.7698460 -0.6305900

H 2.4237820 -1.7365440 0.9904360

H 2.6172330 -2.7685950 -2.3038300

C 3.6457590 -3.6683260 -0.5674000

C 2.0155470 -2.7500200 1.0178630

C 2.4521760 -3.5141940 -1.5257490

H 3.8904450 -3.6577460 1.6073780

C 3.1019050 -3.8117840 0.8676210

H 4.2557880 -4.5275930 -0.8531240

H 1.3280180 -2.8533410 1.8618470

H 2.1228730 -4.4588270 -1.9637660

H 2.6804760 -4.8096030 1.0290980

H 5.3947620 2.6982230 -1.6601410

H 4.4748660 0.8537340 -3.4241080

H 4.0430980 1.8614950 0.1775570

C 4.3633950 3.0657200 -1.6662370

C 3.5370970 1.3904330 -3.2575180

C 3.5099400 2.2483950 -0.6901990

H 4.4197240 3.2809000 -3.8467950

C 3.7583710 2.8911600 -3.0702490

H 4.3829010 4.1144480 -1.3628130

H 2.8063230 1.1174940 -4.0200540

H 2.6207640 2.7822450 -0.3494620

H 2.8033210 3.4217750 -3.1431170

H -4.4730560 3.8232330 -0.3617240

H -2.1320840 5.2769110 -0.6440580

H -3.4879290 2.5376390 1.3930360

C -3.8490240 3.0085080 -0.7427490

C -1.7778880 4.3152450 -1.0252410

C -3.0403540 2.3922550 0.4079610

H -3.3584530 4.2129430 -2.5023500

C -2.8594370 3.5539660 -1.7886070

H -4.4985590 2.2533940 -1.1878510

H -0.8358340 4.4459950 -1.5598890

H -2.7982390 1.3325940 0.2748610

H -2.4099250 2.7275520 -2.3433400

H 2.2746680 1.4917010 5.2112790

H 0.1371200 2.6897090 3.1494380

H 3.2953190 -0.1609450 3.6519480

C 1.6649100 0.6189050 4.9556670

C 0.1575130 1.6002580 3.2037490

C 2.2103440 -0.0518660 3.6954640

H -0.1617640 1.7613450 5.3504180

C 0.2124330 1.0286910 4.6326490

H 1.6974650 -0.0744560 5.7985440

H -0.6548200 1.1937040 2.6000520

H 1.6863400 -0.9909290 3.4939090

H -0.4136670 0.1348970 4.6695030

Mg -0.0456680 0.2794270 -0.3592210

S -1.3338340 -1.5581740 1.9987160

O -0.3811730 -1.7517980 3.2023580

C -2.3833960 -3.0926130 1.6014610

O -1.5152260 -0.3917020 0.7969850

C -2.7669670 -0.8499170 2.9151630

C -4.0101360 -1.2923940 2.1663570

C -3.8483370 -2.8090080 1.9437220

H -4.5158060 -3.1673150 1.1541280

H -4.1214420 -3.3354130 2.8643240

H -4.9202890 -1.0710640 2.7311410

H -4.0701940 -0.7650210 1.2085950

H -2.1976740 -3.3236990 0.5532460

H -1.9227700 -3.8774820 2.2046260

H -2.6451180 0.2324330 2.9800880

H -2.7305980 -1.2848090 3.9187260

O -1.3705210 0.4652070 -2.0111430

O -3.7666690 0.3896980 -2.7361300

S -2.4832570 -0.3005260 -2.6323730

H -0.2102200 -1.9797080 -3.3666820

H -2.0076060 -1.8352790 -0.9289330

H -1.2309760 -0.1333160 -4.6464750

C -1.1703030 -2.2056660 -3.8398970

C -2.6185290 -1.9138190 -1.8289100

C -1.8736800 -0.9023400 -4.2153340

H -1.5634380 -3.7356370 -2.3254180

C -2.0953530 -2.9374440 -2.8473770

H -0.9820470 -2.8095940 -4.7300270

H -3.6657940 -2.0479420 -1.5525500

H -2.7472010 -1.0678920 -4.8521980

H -2.9351430 -3.3826120 -3.3896430

91

TMS_6_Mg^2+^ C-S dissociated G=-4434.475053

O 0.5071560 4.1181000 0.0867590

S -0.6415500 3.2235790 -0.0532350

O -0.3650610 1.9067730 -0.6809590

O -3.0667660 -1.8929010 2.2598340

O -1.6465560 -0.1706140 1.1300130

S -1.9184380 -0.9898030 2.3470770

O -3.5686220 1.2321900 -1.0238960

O -1.7252630 -0.3058950 -1.8166380

S -3.1755340 -0.0417980 -1.6249400

O 0.0740980 -4.4850240 0.8521760

S 0.5293530 -3.5214830 -0.1467800

O -0.2962970 -2.2926960 -0.2956320

H -3.8273270 3.5005260 0.0287020

H -2.0542370 2.0864680 1.4292290

H -2.3033460 3.4151300 -1.7521980

C -3.0830180 4.2954800 0.1181060

C -1.5072190 3.0300530 1.5054360

C -1.9850420 4.0617210 -0.9342380

H -3.1973730 4.1292270 2.2974790

C -2.4399370 4.2380560 1.5183020

H -3.5792650 5.2524130 -0.0554700

H -0.7588530 2.9622930 2.2984610

H -1.5505700 4.9856060 -1.3218070

H -1.8727900 5.1513500 1.7269070

H -5.8906020 -1.5441800 -1.6867650

H -4.8140380 0.3763350 -3.2732490

H -4.3444290 -1.1406420 0.1467400

C -4.9216540 -2.0397890 -1.8064000

C -3.9521630 -0.2950740 -3.2340190

C -3.9158900 -1.4750430 -0.7974510

H -5.1150240 -1.9616670 -3.9884320

C -4.3687140 -1.7655920 -3.2158740

H -5.0710260 -3.1072850 -1.6320760

H -3.2280790 -0.0236130 -4.0034520

H -3.0906710 -2.1590840 -0.5889090

H -3.5008920 -2.4025160 -3.4164730

H 3.3112860 -4.7220250 -0.8444870

H 0.7361710 -5.3518390 -1.6201410

H 2.5682490 -3.4350970 1.0622440

C 3.0066350 -3.7014380 -1.0982270

C 0.7086410 -4.2706410 -1.7793580

C 2.2664960 -3.0622670 0.0816630

H 2.4182320 -4.3439530 -3.1079400

C 2.0390950 -3.7223830 -2.2940130

H 3.8994420 -3.1190050 -1.3297310

H -0.1656210 -3.9994970 -2.3729540

H 2.3066780 -1.9701570 0.0756070

H 1.8989870 -2.7076970 -2.6747760

H -2.3894840 -1.5942470 5.1187090

H -0.6588380 -2.9366450 2.7419880

H -3.1185110 0.4132080 3.8419380

C -1.6203300 -0.8440830 4.9082280

C -0.4580780 -1.8806520 2.9313640

C -2.0828040 0.0751100 3.7788030

H -0.0731090 -2.3895650 5.0144850

C -0.3150810 -1.5068960 4.4191500

H -1.4504450 -0.2698080 5.8212980

H 0.3822450 -1.5354380 2.3259470

H -1.3836210 0.9018770 3.6290740

H 0.4950940 -0.7801750 4.5069640

Mg -0.1772370 -0.1774210 -0.3931070

S 1.7795280 1.0284180 1.8927950

O 0.7366230 1.1291260 2.9813650

C 4.9833710 2.2073360 1.2037920

O 1.3790370 -0.0824120 0.8561620

C 3.1404640 0.1192630 2.7017120

C 4.3252240 -0.1998760 1.7854530

C 5.4152510 0.8830090 1.7338090

H 6.2549530 0.5063610 1.1375180

H 5.8165810 1.0092060 2.7563590

H 4.7888840 -1.1258280 2.1443680

H 3.9641990 -0.4119460 0.7718610

H 5.7048790 2.8684500 0.7334710

H 4.0268440 2.6377250 1.4862930

H 2.6600770 -0.7938270 3.0724420

H 3.4421990 0.7179740 3.5684340

O 1.1559090 -0.2659740 -2.0190250

O 3.6332410 -0.6749380 -2.0396370

S 2.5286680 0.2560650 -2.2677440

H 0.8634220 2.2785100 -3.5009460

H 1.9757420 1.9398080 -0.6935060

H 1.8625170 0.2654490 -4.5502220

C 1.9346150 2.3308940 -3.7219360

C 2.7730350 1.8385080 -1.4262380

C 2.4839330 0.9216160 -3.9391730

H 2.2109910 3.8035040 -2.1231430

C 2.7030140 2.9134770 -2.5206860

H 2.0678000 2.9391650 -4.6189390

H 3.7328540 1.7805910 -0.9049440

H 3.5116420 0.9297040 -4.3132800

H 3.7135260 3.1936140 -2.8335930

47

G2_2_Mg^+^ G=-1124.994364

O 2.2929100 1.1603930 -0.5887950

C 3.6145480 0.6359700 -0.5931950

H 4.2664120 1.2600970 -1.2144520

C 3.5206130 -0.7643620 -1.1598020

H 4.0091540 0.6215580 0.4309430

C 2.1418460 2.4420760 0.0032530

H 2.8349120 3.1578850 -0.4529320

C 0.7169480 2.8708190 -0.2586190

H 0.5692690 3.0800300 -1.3258120

H 0.4864580 3.7734370 0.3191890

O -0.1459280 1.8068140 0.1349820

C -1.5214450 2.1769790 0.0323920

H -1.7564260 2.4843820 -0.9937150

H -1.7339040 3.0013310 0.7215620

H -2.1188100 1.3043430 0.2969540

H 2.3529350 2.3795480 1.0782320

Mg 0.5635520 -0.2016830 -0.6317730

C -4.9887440 0.7637710 -0.3044300

H -4.5854940 1.6801020 -0.7610810

H -5.3906250 1.0005280 0.6819040

H -5.7969520 0.3813060 -0.9430140

O -3.9797420 -0.2042030 -0.1218330

C -3.3681940 -0.5832630 -1.3301320

H -2.9040260 0.2881210 -1.8196850

H -4.1016480 -1.0110430 -2.0320550

C -2.3085120 -1.6209860 -1.0353350

H -1.7567800 -1.8555800 -1.9496260

H -2.7507560 -2.5390960 -0.6363590

O -1.3519090 -1.1214750 -0.0910490

C -1.5820630 -1.5310160 1.2584350

H -1.3414870 -2.5964760 1.3532880

H -2.6270780 -1.3611500 1.5287240

C -0.6731420 -0.6984490 2.1290200

O 2.4724390 -1.4198700 -0.4563720

H 4.4685790 -1.2938880 -1.0094500

H 3.2860670 -0.7388140 -2.2320500

C 2.3970970 -2.8179350 -0.7187350

H 3.3268630 -3.3060610 -0.4078480

H 2.2131630 -3.0037630 -1.7829440

H 1.5661600 -3.2032150 -0.1266530

H -1.0189190 0.3399090 2.2026750

O 0.6180700 -0.7244550 1.5303100

H -0.6295130 -1.1279420 3.1365190

C 1.6271140 -0.2087450 2.3875600

H 1.4373290 0.8474610 2.6127430

H 2.5743030 -0.3284640 1.8653230

H 1.6550830 -0.7811530 3.3204790

47

TS1_G2_ G=-1124.978903

C -0.8395100 2.2035400 1.8732870

O 0.1531660 1.9033770 0.9012330

H -1.8036260 1.9958510 1.4072360

H -0.7941360 3.2616230 2.1504350

H -0.7027560 1.5783970 2.7637280

C 1.4806190 2.2119090 1.3290100

H 1.5520610 3.2845490 1.5469530

C 2.4132610 1.8259850 0.2001710

H 1.7075550 1.6472940 2.2427690

H 2.2801110 2.5014900 -0.6573340

O 2.0716640 0.4981160 -0.1781050

H 3.4567040 1.8726490 0.5349420

C 2.8066350 -0.0511670 -1.4107750

H 2.7590120 0.7657930 -2.1427090

C 2.1408120 -1.2799470 -1.9158410

H 3.8216180 -0.2238120 -1.0377350

O 0.8681280 -1.6341540 -1.1061260

H 2.7536440 -2.1806540 -1.8065760

H 1.7665480 -1.1449500 -2.9393800

C 0.1765760 -2.7425000 -1.6784760

H 0.8226130 -3.6280970 -1.6692770

H -0.1224730 -2.5165990 -2.7113490

H -0.7156180 -2.9341290 -1.0811520

H 1.6084480 -1.7352270 3.1737710

C 1.5805610 -1.4879620 2.1071550

H -0.7476960 -2.2014210 3.0732320

H 1.7986880 -2.3756330 1.5030690

H 2.3076940 -0.7150730 1.8706710

C -0.7270220 -1.9258960 2.0116950

H -0.5351110 -2.8290180 1.4168310

O 0.2935590 -0.9704770 1.7677910

C -2.0411530 -1.3013720 1.6172900

H -2.8443390 -2.0452550 1.6599000

H -2.2954980 -0.4614530 2.2763580

O -1.8828130 -0.8237150 0.2846100

C -3.0686960 -0.2729050 -0.2841140

H -3.4950840 0.4633100 0.4090490

H -3.8025990 -1.0663670 -0.4614930

O -1.5612410 1.2340320 -1.2479230

C -2.6594530 0.3934440 -1.5762700

H -2.3508010 -0.3447460 -2.3296220

C -1.2216090 2.1676680 -2.2724960

H -0.9154410 1.6430870 -3.1844840

H -0.3856910 2.7544930 -1.8905360

H -3.4911440 0.9861050 -1.9737620

H -2.0804000 2.8183380 -2.4711370

Mg 0.0551570 0.0606030 -0.2540050

47

TS2_G2_ G=-1125.018486

C -1.5293120 2.5937980 -1.1043480

O -0.2692250 1.9552460 -1.0029330

H -2.2418970 1.8471620 -1.4580020

H -1.4862250 3.4125220 -1.8307630

H -1.8480000 2.9829150 -0.1293710

C 0.7897820 2.8561790 -0.6670280

H 0.8384070 3.6590800 -1.4129330

C 2.0685250 2.0339030 -0.6353360

H 0.5856500 3.2925420 0.3204120

H 2.3527060 1.7648320 -1.6675530

O 1.8228930 0.9077160 0.1453280

H 2.8779140 2.6557230 -0.2233210

C 3.3381840 -0.2800060 0.4306100

H 3.7277940 -0.1866470 -0.5786490

C 2.8563800 -1.5289370 0.8594650

H 3.7599190 0.4007180 1.1607160

O 0.8741210 -1.6085150 0.7275250

H 2.8601670 -1.7503880 1.9214190

H 2.9387140 -2.3832730 0.1973640

C 0.4056100 -2.6616870 1.5047140

H 0.5263530 -2.4730400 2.5855560

H 0.9313440 -3.6021230 1.2745470

H -0.6645950 -2.8303650 1.3128370

H -0.5196250 1.6904580 3.5790260

C -0.0350700 1.0231830 2.8593740

H -2.6966860 0.6939380 2.7907660

H 0.2245630 0.0731180 3.3402670

H 0.8652830 1.4815420 2.4515610

C -2.0926980 0.0932830 2.1012180

H -1.8280330 -0.8545290 2.5875780

O -0.9032810 0.7902200 1.7491930

C -2.8554310 -0.1696290 0.8190250

H -3.6795700 -0.8674490 1.0026040

H -3.2580040 0.7572430 0.3931740

O -1.9091000 -0.7431160 -0.0726080

C -2.3776430 -1.2093970 -1.3314800

H -2.7796040 -0.3726670 -1.9161190

H -3.1621190 -1.9608110 -1.1912910

O -0.1414190 -0.8401400 -1.9908750

C -1.1705070 -1.8221550 -2.0086060

H -0.8363320 -2.7115100 -1.4589310

C 1.0867170 -1.3074890 -2.5428140

H 1.4532980 -2.1662510 -1.9702660

H 1.7966600 -0.4832350 -2.4650090

H -1.4112210 -2.1013520 -3.0407560

H 0.9477700 -1.5784630 -3.5941880

Mg 0.0855200 0.0160620 -0.0112070

47

G2_2_Mg^2+^ O1-C2 dissociated G=-1125.046969

C 2.5726990 1.2941330 1.4084900

O 1.4051100 1.6032760 0.6754400

H 2.2852010 0.5943820 2.1957290

H 2.9859930 2.1965760 1.8723030

H 3.3345700 0.8404920 0.7604770

C 1.6137880 2.5849530 -0.3495170

H 2.0655840 3.4808880 0.0953380

C 0.2422730 2.8636230 -0.9617530

H 2.3042050 2.1665070 -1.0961850

H -0.3271750 3.4808310 -0.2374020

O -0.4105750 1.6887080 -1.2720270

H 0.4051060 3.5055360 -1.8458190

C -3.2924790 0.9403930 -0.7744840

H -4.1709720 0.7652790 -0.1622940

C -2.7308940 -0.1471180 -1.6050960

H -2.7958850 1.9022490 -0.7591230

O -1.7546160 -0.9223310 -0.8393930

H -2.2156440 0.2514240 -2.4838090

H -3.5127960 -0.8486240 -1.9168630

C -1.5293270 -2.2037680 -1.4153750

H -1.0660200 -2.1130210 -2.4061690

H -2.4752900 -2.7471590 -1.5089660

H -0.8709670 -2.7466280 -0.7386350

H 2.6091030 -0.3008860 -2.9628080

C 1.5542740 -0.4134760 -2.6924350

H 3.0038940 -2.0922920 -1.2503260

H 1.0997710 -1.2051060 -3.2997580

H 1.0116370 0.5236320 -2.8220710

C 1.9541520 -2.0033180 -0.9476210

H 1.3774660 -2.7873640 -1.4541370

O 1.4396660 -0.7309760 -1.3046080

C 1.8379910 -2.1231610 0.5605520

H 1.9377930 -3.1691060 0.8698830

H 2.6069520 -1.5280330 1.0667800

O 0.5501260 -1.6240440 0.9097390

C 0.2076150 -1.7217010 2.2891630

H 0.9996940 -1.2665320 2.8965610

H 0.0923260 -2.7720900 2.5770010

O -0.8960920 0.3061580 1.8553970

C -1.0933090 -0.9664870 2.4586080

H -1.9240050 -1.4826250 1.9625760

C -1.8391250 1.3024490 2.2398350

H -2.8588250 0.9722600 2.0174870

H -1.6036420 2.1933430 1.6572790

H -1.3205870 -0.8522280 3.5240970

H -1.7357620 1.5162910 3.3082570

Mg -0.0747190 0.2097110 -0.1098310

47

G2_2_Mg^2+^ C3-O2 dissociated G=-1125.045394

C -0.6043750 -0.9143630 2.8240690

O 0.3577350 -0.2782060 1.9931430

H -1.5563650 -0.4148350 2.6472090

H -0.3280390 -0.7967390 3.8765100

H -0.6825780 -1.9790620 2.5756260

C 1.6784740 -0.7734370 2.1924310

H 1.9357150 -0.7427200 3.2571070

C 2.6024070 0.1225460 1.4019350

H 1.7345970 -1.8125360 1.8414750

H 2.6649420 1.1208890 1.8536890

O 2.0423640 0.2237790 0.0997740

H 3.6071320 -0.3111780 1.3518090

C 2.8849640 0.8729130 -0.8860930

H 2.1957310 0.9928390 -1.7341090

C 4.0742180 0.0560380 -1.2137950

H 3.1617400 1.8643110 -0.5058870

O 0.1080490 0.6471490 -1.9268920

H 5.0362520 0.5204530 -1.3943240

H 3.9556110 -0.9992690 -1.4390350

C -0.7341390 0.4734350 -3.0046740

H -0.5121020 -0.4482480 -3.5803010

H -0.6645190 1.3062020 -3.7268520

H -1.8047820 0.4037230 -2.7188880

H 0.5923920 -3.6946600 -1.1852050

C 0.7112640 -2.6072460 -1.1517390

H -1.8205100 -3.3980530 -0.7463800

H 0.6787480 -2.1825760 -2.1607990

H 1.6607010 -2.3517790 -0.6812110

C -1.6220130 -2.3241980 -0.8365100

H -1.6839950 -2.0299050 -1.8913990

O -0.3152300 -2.0262640 -0.3441780

C -2.6183340 -1.5277060 -0.0181480

H -3.6085730 -1.5764800 -0.4838200

H -2.6879150 -1.8954790 1.0124270

O -2.1286330 -0.1966520 -0.0291770

C -2.9633710 0.8610510 0.4185180

H -3.1355160 0.7763590 1.4987180

H -3.9264770 0.8339180 -0.1026600

O -0.8989950 2.0012560 0.6118970

C -2.2126060 2.1310210 0.0729090

H -2.1473370 2.2482910 -1.0159090

C -0.0579250 3.1078600 0.2843330

H 0.1053240 3.1413810 -0.7980560

H 0.8892530 2.9411740 0.8001160

H -2.7136610 3.0030400 0.5082410

H -0.5054820 4.0405860 0.6419040

Mg -0.0495000 0.1336980 -0.1196620

47

TS1’_G2_ G=-1124.969579

C 0.1250450 1.7742040 2.6142140

O -0.5921300 0.6883760 2.0396740

H 1.1852580 1.5391840 2.4995640

H -0.1106430 1.8631580 3.6794250

H -0.1144940 2.7101410 2.0964440

C -2.0025160 0.7782340 2.2045310

H -2.2533310 0.8034530 3.2713380

C -2.6069260 -0.4479300 1.5569260

H -2.3744420 1.6959030 1.7296460

H -2.2998060 -1.3586010 2.0873080

O -2.0970990 -0.4804780 0.2246060

H -3.7003250 -0.3769940 1.5369330

C -2.4888300 -1.6052280 -0.5611750

H -2.1128970 -2.5226540 -0.0893160

C -1.8658800 -1.4044010 -1.9269380

H -3.5811550 -1.6453450 -0.6408060

O -0.4946550 -1.0736130 -1.7323800

H -2.3638930 -0.5892520 -2.4675810

H -1.9499280 -2.3243520 -2.5172130

C 0.2327070 -0.8947780 -2.9426510

H -0.2111320 -0.0891900 -3.5404970

H 0.2389130 -1.8226750 -3.5232650

H 1.2512760 -0.6292030 -2.6577370

H -1.7592740 3.2126520 -1.2785650

C -1.5797280 2.1346830 -1.3565420

H 0.6197750 3.5050730 -1.3920950

H -1.5626620 1.8480490 -2.4163220

H -2.3648500 1.5839590 -0.8431540

C 0.7543430 2.4165490 -1.3719500

H 0.8282730 2.0537040 -2.4061940

O -0.3496350 1.7945870 -0.7292600

C 1.9978670 2.0602270 -0.5959530

H 2.8921160 2.3587870 -1.1546540

H 2.0110950 2.5561790 0.3832530

O 1.9688770 0.6500280 -0.4192920

C 3.1382170 0.1002910 0.2162980

H 3.2881530 0.6010650 1.1806350

H 4.0028050 0.2943620 -0.4315590

O 1.5802110 -1.5503750 0.9612140

C 2.8877250 -1.3765610 0.4029350

H 2.9626210 -1.8997290 -0.5614730

C 1.2235700 -3.1710770 1.2615320

H 1.1878390 -3.6709270 0.2965980

H 0.2663530 -3.1303000 1.7750970

H 3.6422200 -1.7943220 1.0820590

H 2.0703710 -3.4463180 1.8886690

Mg 0.1078050 -0.3116060 0.2208000

47

G2_2_Mg^2+^ C1-O1 dissociated G=-1125.04354

C 0.6116850 -2.6823970 -1.6896620

O -0.3897800 -1.8604490 -1.1085420

H 1.3293250 -2.0124310 -2.1654210

H 0.1711030 -3.3328100 -2.4518610

H 1.1107150 -3.2860400 -0.9230150

C -1.4851300 -2.5743460 -0.5557530

H -1.9575630 -3.1990900 -1.3223810

C -2.4629940 -1.5373530 -0.0508510

H -1.1365820 -3.2177390 0.2633800

H -2.8927910 -0.9579790 -0.8764400

O -1.7027710 -0.6657940 0.7881980

H -3.2648330 -2.0097620 0.5262010

C -2.4425230 0.3973080 1.3920670

H -2.8893090 1.0161950 0.6028580

C -1.4451160 1.1797870 2.2164070

H -3.2319300 -0.0111700 2.0321320

O -0.3317020 1.4552930 1.3757560

H -1.1110090 0.5998810 3.0869720

H -1.8980720 2.1146030 2.5652800

C 0.6371110 2.3134380 1.9666130

H 1.0405040 1.8632850 2.8823430

H 0.1866190 3.2825990 2.2026880

H 1.4299850 2.4423240 1.2309400

H 1.6648620 -2.4601790 2.5215570

C 1.0531610 -1.5817060 2.2937310

H 3.4799710 -1.3451900 1.3082970

H 1.1601740 -0.8420960 3.0965770

H 0.0065600 -1.8682230 2.1984610

C 2.7889390 -0.5335600 1.0548990

H 2.8803480 0.2593530 1.8082070

O 1.4469650 -1.0188350 1.0460160

C 3.0800690 0.0045450 -0.3292300

H 4.0275240 0.5553650 -0.3329050

H 3.1460250 -0.8109360 -1.0624050

O 1.9944580 0.8538000 -0.6478360

C 1.9777150 1.4166510 -1.9731240

H 2.1477120 0.6051550 -2.6937930

H 2.7789260 2.1600770 -2.0589400

O -0.3807790 1.0717990 -1.7924700

C 0.5733310 2.0025850 -2.1422300

H 0.5203660 2.9225630 -1.5254930

C -3.5596390 1.6923860 -1.6961840

H -3.9504930 2.5805630 -1.2103690

H -4.2657150 0.9964860 -2.1375410

H 0.4844950 2.3303810 -3.1928930

H -2.4877310 1.5746810 -1.8563690

Mg 0.0391710 0.0649660 -0.2270950

49

G1_3_Mg^+^ G=-1126.178952

O 2.1702440 1.3436090 -0.9127440

C 3.5250650 0.9175040 -0.9665900

H 4.0865060 1.5275580 -1.6814610

H 3.5148040 -0.1218760 -1.2947280

H 3.9917260 0.9928280 0.0237000

C 2.0064480 2.6831340 -0.4712960

H 2.5638000 3.3673840 -1.1221320

C 0.5325180 3.0099360 -0.5389930

H 0.1944910 3.0591530 -1.5818940

H 0.3413740 3.9720040 -0.0502870

O -0.1876790 1.9747740 0.1332410

C -1.5825640 2.2741730 0.2323150

H -1.9920340 2.4913370 -0.7612670

H -1.7259450 3.1371240 0.8903750

H -2.0825760 1.3971770 0.6421370

H 2.3893820 2.7849230 0.5522940

Mg 0.4310950 0.0085760 -0.6772760

C -4.9348350 0.7649960 -0.1732960

H -4.5129960 1.6140920 -0.7316330

H -5.3819180 1.1297300 0.7527660

H -5.7126230 0.2937740 -0.7903340

O -3.9343660 -0.1616840 0.1833360

C -3.2692980 -0.6922620 -0.9360900

H -2.7898800 0.1096640 -1.5223630

H -3.9715120 -1.2218230 -1.6006260

C -2.2069400 -1.6679750 -0.4795340

H -1.6871770 -2.0564180 -1.3606880

H -2.6451130 -2.5040630 0.0756100

O -1.2119990 -1.0401080 0.3401560

C -1.5032320 -1.1109250 1.7409620

H -1.3979180 -2.1447440 2.0894140

H -2.5155520 -0.7514130 1.9312490

H -0.7745780 -0.4830060 2.2489350

O 1.4245330 -0.3618570 1.3231930

C 2.2622240 -1.5067950 1.4054560

C 1.7438930 -2.5281890 0.4229790

O 1.5922450 -1.8674240 -0.8284130

C 1.3555480 -2.7696290 -1.9100390

H 1.1659190 -2.1574810 -2.7922110

H 2.2373890 -3.3999940 -2.0626140

H 0.4814170 -3.3970530 -1.7012220

H 2.4604100 -3.3526690 0.3335580

H 0.7741520 -2.9307010 0.7414340

H 3.2944330 -1.2220130 1.1636160

H 2.2398210 -1.9200930 2.4211550

C 1.7407770 0.6203530 2.3013970

H 2.7725580 0.9709230 2.1768190

H 1.0453750 1.4458730 2.1566090

H 1.6216780 0.2024910 3.3073320

49

TS1_G1_ G=-1126.158959

C -1.5029590 1.7842720 2.0956700

H -1.0153630 2.0589930 3.0382860

H -1.9704950 0.8038290 2.1824190

H -2.2540590 2.5358260 1.8330940

O -0.5392950 1.6722690 1.0522120

C 0.2172800 2.8454460 0.7936740

H 1.1299710 2.8343380 1.4001490

H -0.3614770 3.7384940 1.0526150

C 0.5369150 2.8676760 -0.6852260

H 1.3228850 3.6039340 -0.8907210

H -0.3535620 3.1338440 -1.2686660

O 0.9764560 1.5675060 -1.0629560

C 1.3909270 1.5112480 -2.4280410

H 2.2196530 2.2081950 -2.5910720

H 0.5561550 1.7635760 -3.0922880

H 1.7165640 0.4893550 -2.6119250

O 1.3303470 -1.4223320 -1.2236550

C 2.4017640 -1.9181260 -0.4294760

C 2.8237290 -0.8084030 0.4995350

O 1.6607140 -0.3651170 1.1924550

C 1.9668870 0.3595830 2.3757540

H 1.0214790 0.6953810 2.8002150

H 2.4818670 -0.2906250 3.0913330

H 2.6022010 1.2237100 2.1492290

H 3.5626480 -1.1892290 1.2139480

H 3.2597950 0.0330340 -0.0530260

H 2.0617040 -2.7916990 0.1416520

H 3.2420270 -2.2147380 -1.0687020

C 0.8933410 -2.3594810 -2.2109180

H 0.5677200 -3.2915220 -1.7343070

H 0.0398920 -1.8996210 -2.7155830

H 1.7106780 -2.5535540 -2.9150420

H -0.3826690 -3.0911930 1.8304420

H -2.0128170 -2.9414610 0.0989220

H -2.6860350 -1.6679200 -1.6886450

H -2.2328960 0.3531280 -2.9140710

Mg -0.1389060 -0.1103370 -0.1193530

C -0.7792340 -2.1479520 2.2285660

C -2.4070050 -1.9686250 0.4234010

C -2.8686870 -1.1524420 -0.7345850

C -2.4989180 0.8947080 -1.9958480

H 0.0131460 -1.6151620 2.7519150

O -2.0983950 0.1518430 -0.8432400

O -1.2497210 -1.3172740 1.1669840

H -1.9876620 1.8572310 -1.9772280

H -1.5998540 -2.3613750 2.9226570

H -3.1771170 -2.0805520 1.1952200

H -3.9106550 -0.8252440 -0.6344350

H -3.5819350 1.0624850 -1.9635810

49

G1_3_Mg^2+^ O1-C2 dissociated G=-1126.229357

C -0.3505460 -1.9069200 2.6197440

H -1.3934680 -1.8175400 2.9458340

H 0.2258360 -1.0551580 2.9795270

H 0.0660990 -2.8564400 2.9700750

O -0.2791260 -1.8513640 1.1924670

C -1.1648980 -2.7473750 0.5379570

H -2.2000360 -2.4279550 0.7130950

H -1.0310860 -3.7648630 0.9230500

C -0.8317260 -2.7088340 -0.9350780

H -1.6032690 -3.2411780 -1.5024310

H 0.1421490 -3.1729470 -1.1345500

O -0.7866830 -1.3409150 -1.3363650

C -0.8698180 -1.1718500 -2.7477250

H -1.8629290 -1.4684440 -3.1021120

H -0.1088410 -1.7756960 -3.2544010

H -0.7019240 -0.1144460 -2.9507730

O -0.3104630 1.6164970 -1.1990170

C -1.5648930 2.2868630 -1.0772460

C -2.5953540 1.2431830 -0.6898220

O -2.1595080 0.5266870 0.4605350

C -2.5283140 1.1412530 1.6992250

H -2.0480880 0.5697080 2.4895970

H -2.1647750 2.1719230 1.7526790

H -3.6176620 1.1153710 1.8030300

H -3.5670450 1.7139550 -0.5074800

H -2.7113620 0.5041880 -1.4850170

H -1.4779770 3.0706040 -0.3137710

H -1.8333520 2.7536930 -2.0323620

C 0.7622760 2.4974430 -1.5342670

H 0.8447610 3.2935360 -0.7872640

H 1.6723410 1.8994610 -1.5304880

H 0.5939170 2.9279040 -2.5263840

H 0.1834770 2.9414550 1.5298410

H 5.0954470 0.2047190 0.6510230

H 3.2353480 -1.4345030 0.7502090

H 3.3985480 -0.5521760 -1.8981020

Mg -0.0428840 0.0190470 0.2217390

C 0.8846030 2.2069460 1.9828290

C 4.0976770 0.4896880 0.3391270

C 2.9375430 -0.3872850 0.6150740

C 2.3868020 -0.8977200 -1.6634320

H 0.9638970 2.4858470 3.0475610

O 1.9472220 -0.3208970 -0.4414040

O 0.4842420 0.8979270 1.8183410

H 1.7068980 -0.5665760 -2.4484530

H 1.8777420 2.4168660 1.5333430

H 3.9312100 1.5036200 -0.0117050

H 2.3719230 -0.0570020 1.4988290

H 2.3812510 -1.9923160 -1.5980440

61

G3_2_Mg^+^ G=-1432.546504

C -1.8239230 -0.2540270 1.7383750

O -1.1213210 -0.8498520 0.6536840

H -1.5125810 0.7848730 1.7837010

H -1.5750620 -0.7727520 2.6753450

H -2.8982810 -0.2983900 1.5483120

C -1.5202660 -2.1977940 0.4595070

C -0.6988930 -2.7739330 -0.6668420

H -2.5860340 -2.2344620 0.2092190

H -1.3405380 -2.7653440 1.3841280

O 0.6561950 -2.4776950 -0.3610070

H -0.8506710 -3.8585830 -0.7300570

H -0.9647310 -2.3112060 -1.6276250

C 1.6000880 -3.0380910 -1.2576640

H 1.5548350 -4.1337060 -1.2285940

C 2.9528500 -2.5471890 -0.7960620

H 1.3956630 -2.6903660 -2.2793440

Mg 0.7859230 -0.0685670 -0.4092540

H -3.4349500 1.5967450 -2.4007040

H -2.5653930 0.1295560 -1.8810870

H 1.9453460 2.7036060 1.1715940

C -3.2272820 0.9403410 -1.5411710

H -0.9354180 1.9561450 -1.7119480

H -0.1096210 3.8372280 -0.1866550

C 1.2917900 1.1198130 2.4962930

O -2.6013210 1.6742390 -0.5099120

C 1.0245220 2.3308590 1.6299560

O 0.1143670 1.9376240 0.6099290

C -1.5786440 2.5036850 -1.0018600

C -0.7277570 2.9926330 0.1446670

H 0.5867940 3.1195460 2.2527310

H -2.0051430 3.3758230 -1.5238810

H -1.3656380 3.3325600 0.9703850

O 1.5526550 0.0003620 1.6581650

C 1.6902010 -1.2017680 2.4082320

O 2.8464060 -1.1376770 -0.7116970

C 4.0140520 -0.4378340 -0.3102980

H 4.9150350 -0.9895220 -0.6006990

H 4.0079390 -0.3071260 0.7779380

H 0.8063050 -1.3601840 3.0371050

H 2.5846150 -1.1512410 3.0387270

H 1.7689760 -2.0197250 1.6961870

H 0.4196910 0.8968050 3.1229910

H 2.1533020 1.3057030 3.1494040

H 3.7282920 -2.8315000 -1.5176260

H 3.2104260 -2.9716140 0.1838310

C -4.5459510 0.3790370 -1.0620850

H -5.1152480 0.0238430 -1.9362240

H -5.1291660 1.1796130 -0.5815920

C -5.5378230 -1.2191160 0.3317090

H -6.1589460 -1.6256880 -0.4799490

H -5.2749160 -2.0264940 1.0186550

H -6.1218350 -0.4616360 0.8742420

C 3.9812930 0.8883100 -1.0308410

H 4.8059270 1.5315260 -0.6949620

H 4.0842190 0.7164740 -2.1108380

C 2.5128510 2.6257590 -1.6007370

H 1.5269580 3.0192560 -1.3581830

H 3.2730870 3.3956520 -1.4193570

H 2.5276280 2.3337590 -2.6576160

O 2.7333180 1.5025410 -0.7647150

O -4.3333740 -0.6833510 -0.1623950

61

TS1_G3_ G=-1432.527935

O -1.6537360 -0.1797510 1.0708110

C -1.3254540 0.5054100 2.2709750

C -0.2475450 -0.3155590 2.9435320

H -2.1984710 0.5952340 2.9231900

H -0.9743840 1.5131890 2.0314820

O 0.7478440 -0.5439590 1.9504600

H -0.6477960 -1.2775460 3.2883310

H 0.1885300 0.2214390 3.7939620

C 1.8574510 -1.3328710 2.3715000

C 2.8626910 -1.2805280 1.2449320

H 1.5215370 -2.3599710 2.5627540

H 2.2873060 -0.9172550 3.2897330

O 2.1496410 -1.5916230 0.0526940

H 3.6546510 -2.0192710 1.4168630

H 3.3072390 -0.2829620 1.1513650

C 2.9946930 -1.6850950 -1.0966150

H 3.7129470 -2.5009140 -0.9594360

H 3.5078500 -0.7327160 -1.2577540

H 2.3248220 -1.8985170 -1.9364430

C -3.0368550 -0.2924990 0.7395340

C -3.5576540 0.9109680 -0.0155510

H -3.6039820 -0.4481140 1.6643510

H -3.1185150 -1.1840380 0.1138820

O -3.2569090 2.0759020 0.7145140

H -4.6460320 0.7948610 -0.1464910

H -3.0994840 0.9468960 -1.0138060

C -3.7942320 3.2361630 0.1235870

H -4.8918040 3.1919710 0.0814980

H -3.4120110 3.3801930 -0.8986800

H -3.4921460 4.0839550 0.7409410

Mg 0.0828410 -0.8385910 -0.1473560

C -1.3316270 -2.7692830 -1.9221900

H -2.3097990 -3.2638350 -1.9753560

H -0.5098250 -3.4423880 -2.2104590

O -1.1290680 -2.5169150 -0.4521970

C -1.1242800 -3.7171370 0.3185760

H -1.0318030 -3.4337240 1.3687110

H -2.0667860 -4.2570560 0.1714040

H -0.2807490 -4.3575960 0.0315080

C -1.2843400 -1.5003870 -2.7107650

H -2.2256760 -1.2763040 -3.2262130

H -0.4147560 -1.4906540 -3.3849080

O -1.0788760 -0.3041960 -1.8007320

C -0.6517470 0.8840150 -2.4603960

H -1.4561390 1.2740580 -3.0970480

H 0.2286080 0.6579980 -3.0799810

C -0.2911730 1.8953340 -1.3949490

H -1.1735900 2.2407010 -0.8421110

H 0.2059220 2.7569090 -1.8554650

O 0.5979200 1.2594310 -0.4692290

C 1.2882900 2.1664390 0.3930980

H 0.6312770 3.0105650 0.6363320

H 1.5002650 1.6114220 1.3079650

C 2.5835610 2.6632370 -0.2108380

H 2.4119580 3.1002730 -1.2073740

H 2.9859330 3.4612020 0.4341480

O 3.4820150 1.5866360 -0.2902360

C 4.7293540 1.9575020 -0.8330420

H 5.3532070 1.0617320 -0.8470700

H 4.6212000 2.3374890 -1.8586680

H 5.2189680 2.7273810 -0.2198970

61

G3_2_Mg^2+^ O1-C2 dissociated G=-1432.595169

C 2.0344640 -0.1629350 2.3632230

O 1.6818500 0.6549940 1.2527000

H 1.1149900 -0.6315350 2.7093810

H 2.4567780 0.4532090 3.1642120

H 2.7496670 -0.9255260 2.0421860

C 2.7365410 1.4871470 0.7816820

C 2.6014820 1.6059450 -0.7214820

H 3.7062570 1.0412240 1.0240970

H 2.6583130 2.4653280 1.2705070

O 1.2181470 1.8216210 -0.9859700

H 3.1950530 2.4522990 -1.0850600

H 2.9420980 0.6837740 -1.2046270

C 0.8900000 2.1779060 -2.3665950

H 1.8028810 2.5670850 -2.8276510

C -0.1884010 3.1920560 -2.3601070

H 0.5700040 1.2623210 -2.8687360

Mg -0.0824010 0.4601010 0.0430520

H 0.7047320 -2.6309720 -1.9513930

H 1.3956000 -1.0067490 -1.9185600

H -2.9765760 -0.0083120 1.4290990

C 1.2841150 -1.9307720 -1.3421170

H -1.3433010 -2.1457950 -0.6750600

H -2.1447280 -2.3801460 1.5647620

C -1.7022340 1.4643970 2.4048390

O 0.5745480 -1.6194670 -0.1422930

C -2.1585680 0.0446980 2.1546710

O -1.0220900 -0.6313080 1.6193220

C -0.5974700 -2.4065570 0.0801400

C -1.1087670 -2.0481290 1.4629010

H -2.4631130 -0.4148640 3.1016420

H -0.3510360 -3.4740210 0.0394190

H -0.5002400 -2.5080800 2.2484830

O -1.1173940 1.9510500 1.2011040

C -0.6250520 3.2808690 1.3196050

O -1.2202580 0.2256160 -1.4466980

C -2.5222500 0.5915470 -1.6817680

H -2.6412890 1.0882900 -2.6671360

H -2.9086770 1.3124500 -0.9316750

H 0.1492410 3.3324550 2.0948010

H -1.4446160 3.9629100 1.5683810

H -0.1986610 3.5469410 0.3525210

H -0.9522970 1.4940630 3.2069110

H -2.5542280 2.0932610 2.6884050

H -1.2207350 2.8817270 -2.2541520

H 0.0482120 4.2507050 -2.3665060

C 2.6392050 -2.5127210 -1.0124440

H 3.1241720 -2.8441550 -1.9444600

H 2.5223490 -3.3922530 -0.3599600

C 4.7083600 -1.9735230 -0.0523590

H 5.2655690 -2.2690250 -0.9524940

H 5.2277440 -1.1439590 0.4318360

H 4.6738430 -2.8280400 0.6382000

C -3.4862770 -0.5866660 -1.7042870

H -4.4883450 -0.2538200 -2.0236070

H -3.1282760 -1.3397940 -2.4251830

C -4.4439000 -2.2632810 -0.3707360

H -4.4675080 -2.6289520 0.6596340

H -5.4643690 -1.9790680 -0.6675040

H -4.1045930 -3.0755750 -1.0315890

O -3.5683270 -1.1654440 -0.4135400

O 3.4118240 -1.5231250 -0.3754680
